# Nanozyme-based colorimetric biosensor with a systemic quantification algorithm for noninvasive glucose monitoring

**DOI:** 10.7150/thno.72152

**Published:** 2022-09-07

**Authors:** Hee-Jae Jeon, Hyung Shik Kim, Euiheon Chung, Dong Yun Lee

**Affiliations:** 1 Weldon School of Biomedical Engineering, Purdue University, Indiana 47906, USA; 2 Department of Mechanical and Biomedical Engineering, Kangwon National University, Chuncheon 24341, Republic of Korea; 3 Department of Bioengineering, College of Engineering, and BK FOUR Biopharmaceutical Innovation Leader for Education and Research Group, Hanyang University, Seoul 04763, Republic of Korea; 4 Department of Biomedical Science and Engineering, Gwangju Institute of Science and Technology (GIST), Gwangju 61005, Republic of Korea; 5 AI Graduate School, GIST, Gwangju 61005, Republic of Korea; 6 Research Center for Photon Science Technology, GIST, Gwangju 61005, Republic of Korea; 7 Institute of Nano Science and Technology (INST), Hanyang University, Seoul 04763, Republic of Korea; 8 Institute for Bioengineering and Biopharmaceutical Research (IBBR), Hanyang University, Seoul 04763, Republic of Korea; 9 Elixir Pharmatech Inc., Seoul 07463, Republic of Korea

**Keywords:** nanozyme, colorimetric analytic methods, glucose, colorimetric biosensor

## Abstract

Diabetes mellitus accompanies an abnormally high glucose level in the bloodstream. Early diagnosis and proper glycemic management of blood glucose are essential to prevent further progression and complications. Biosensor-based colorimetric detection has progressed and shown potential in portable and inexpensive daily assessment of glucose levels because of its simplicity, low-cost, and convenient operation without sophisticated instrumentation. Colorimetric glucose biosensors commonly use natural enzymes that recognize glucose and chromophores that detect enzymatic reaction products. However, many natural enzymes have inherent defects, limiting their extensive application. Recently, nanozyme-based colorimetric detection has drawn attention due to its merits including high sensitivity, stability under strict reaction conditions, flexible structural design with low-cost materials, and adjustable catalytic activities. This review discusses various nanozyme materials, colorimetric analytic methods and mechanisms, recent machine learning based analytic methods, quantification systems, applications and future directions for monitoring and managing diabetes.

## 1. Introduction

**Figure 1:**
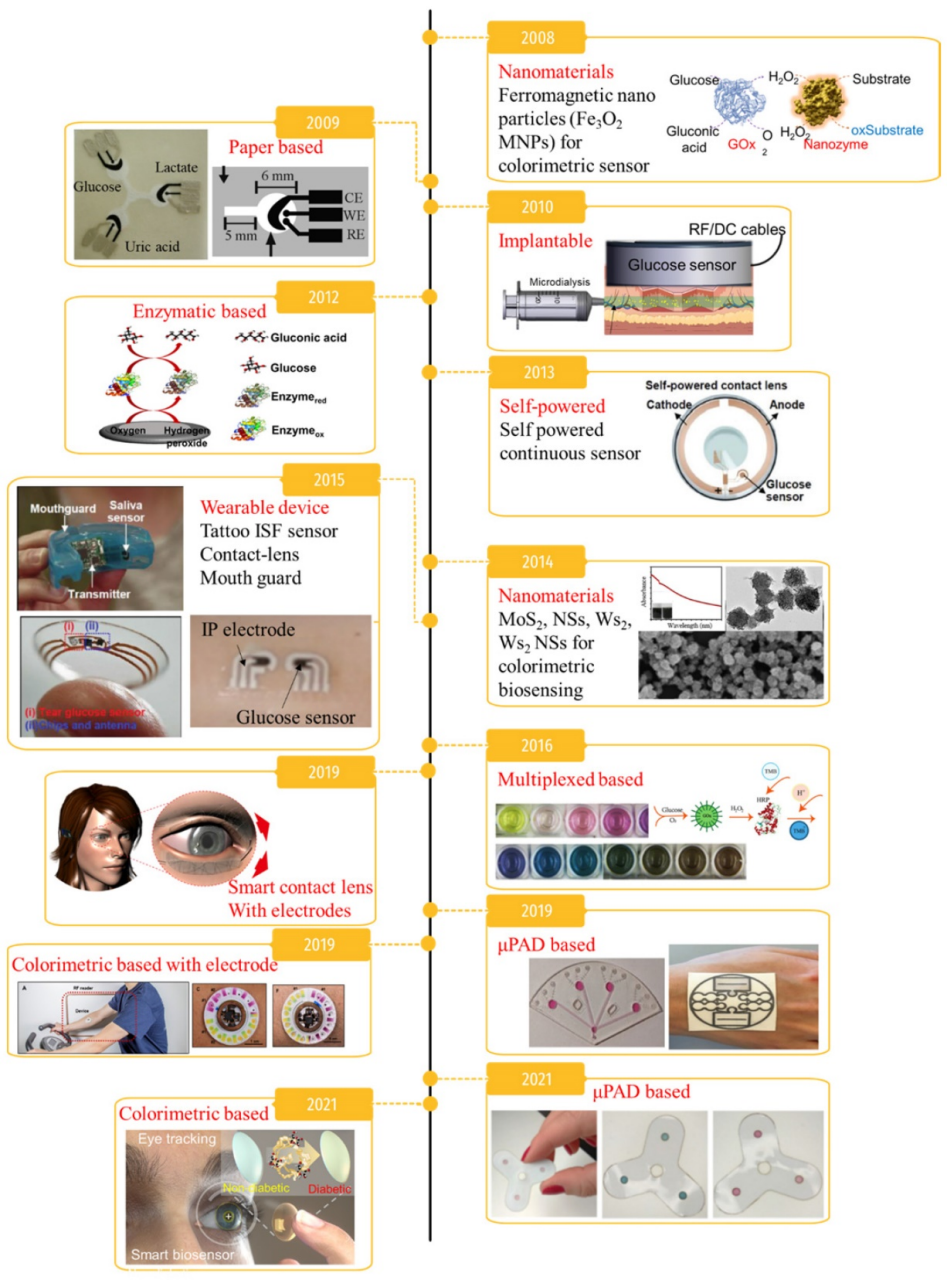
** A brief timeline of glucose monitoring methods and its applications.** Adapted with permission from 116-126.

Diabetes mellitus is a significant cause of death and a chronic disorder affecting more than 422 million people worldwide 1. Chronically elevated blood glucose can cause retinopathy, cardiovascular diseases, neuropathy, blindness, and a high risk of congenital disability 2, causing 1.6 million deaths every year according to the World Health Organization (WHO) 3. Therefore, early diagnosis and proper glycemic management are crucial to prevent further progression and complications in diabetic patients. Millions of diabetic patients rely on self-monitoring of blood glucose, but the current approaches have vast opportunities for improvement. In general, to manage blood glucose in diabetic patients, blood should be collected using a finger-prick method several times every day 4, 5. While direct monitoring of blood glucose is the most accurate measurement 6-8, it can create mental trauma for patients as well as infections and fingertip inflammation due to the requirement of several measurements a day, which is a major issue in terms of patient compliance with glycemic methods. Therefore, the demand for technology development to monitor blood glucose using noninvasive strategies has increased. Nanomaterial-based invasive and noninvasive glucose monitoring methods developed in the last 15 years are summarized in **Figure 1**. Advances in nanotechnology have led to the development of ultra-sensitive and high-performance platforms, including colorimetric, fluorometric, chemiluminescent, surface-enhanced Raman scattering, and electrochemical biosensors 9. In addition, nanomaterial-based glucose monitoring biosensors have expanded from using blood to utilizing bodily fluids including sweat, tears, urine, saliva, and interstitial fluid (ISF).

Conventionally, the basic principle of detecting glucose is based on the glucose oxidase (GO) enzyme, which recognizes glucose as a substrate and initiates a biochemical enzymatic reaction. Therefore, since the reactivity of this enzyme has a significant influence on the sensitivity and reliability of the biosensor, much research is being conducted to improve the performance of the GO enzyme using protein engineering technology. Since the term 'Nanozyme' was first used in 2004, research and development using various functional nanomaterials that mimic the function of the GO enzyme have been conducted 10. The definition of a nanozyme has been solidified into an enzyme-mimicking nanomaterial that demonstrates intrinsic peroxidase-like activity 11, 12. Functional nanomaterials as artificial enzymes (nanozymes) show several remarkable advantages, including simple and excellent tunable catalytic activity, controllable synthesis protocols, ultrahigh environmental stability, ease of modification, low cost, and large-scale production 13, 14. These properties greatly facilitate automation of multiple processes and high-speed integration of separation and detection procedures, saving time and reducing preparation steps. Recently, a few types of inorganic nanoparticles such as nanocarbon materials (carbon nanotubes and graphene oxide) 15, polymer-coated nanoparticles 16, 17, and nanocomposites 17, have been spotlighted for their ability to catalyze chemical reaction as enzyme-mimics that can be utilized for bio-detection. These nanozymes have been applied as chemical sensors and biosensors for colorimetric detection of pH, temperature, ions, reductive small molecules, H_2_O_2_, glucose, viruses, bacteria, cancer cells, and pesticides 18. In addition, invasive and noninvasive biosensors using these nanozymes are being developed to monitor glucose. Over the last decade, colorimetric-based biosensors with nanozymes have expanded due to several advantages including high efficiency, high versatility, low-cost, and high stability.

Nanozyme-based biosensors mainly detect color in a qualitative manner 19, 20. Therefore, for quantitative measurement of glucose concentration, many algorithms have been developed in different color spaces (RGB, CMYK, HSB/HSL, CIE XYZ, L*a*b*, and YUV models) to analyze color variations. In addition, since color values can be affected by the measurement area of the device or the surrounding environment, algorithms including supervised learning based convolutional neural networks (CNN), artificial neural network (ANN), and support vector machine (SVM), polynomial regression, statistical learning-based color reconstruction, and color checker-based digital image reconstruction are being developed to improve these errors 21.

This review highlights important advances in colorimetric-based glucose detection utilizing nanozymes, various mechanisms, colorimetric analysis methods, and their applications in biosensors. This review also discusses sensor performance in whole blood, saliva, urine, tears, and interstitial fluid. Finally, we address future development requirements of biosensing nanozymes for wearable glucose sensors.

## 2. Invasive and noninvasive/minimally invasive glucose detection

Various enzyme-based glucose sensors have been developed for bodily fluids including whole blood, urine, saliva, tear, and interstitial fluid (ISF) 22, 23. The finger-pricking whole blood test is the most widely used method for fast glucose monitoring because blood possesses a high biomolecule concentration 6. However, since this method requires pricking a finger to extract a small amount of blood for analysis up to 8 times a day, procedural discomfort decreases patient compliance. In addition, there is a high risk of complications such as infection and fibrosis. Therefore, implantable biosensors and microdialysis-type devices that can continuously monitor blood glucose have been developed 6. However, implantable glucose sensors pose several challenges, including short lifespan, susceptibility to infection, and poor biocompatibility. Therefore, alternative methods have been explored for noninvasive glucose measurements using body fluids 24.

Several studies have shown that glucose concentrations in body fluids such as ISF, tears, saliva, and sweat correlate with that of whole blood 13. Therefore, many studies have focused on these body fluids to develop noninvasive biosensors for glucose monitoring. To this end, advances in nanotechnology have made it an attractive and alternative sample medium for noninvasive continuous blood monitoring. **Table 1** compares the key aspects of the most studied nanomaterials for various physiological measures, including biomarkers, representative concentrations, and their advantages and disadvantages. However, various considerations are required to protect the quality and accuracy of glucose concentration measurements in body fluids. One of the main considerations is that glucose concentration in these body fluids is lower than in whole blood (2-40 × 10^-3^ M in blood vs. 0.008-1.77 × 10^-3^ M in saliva, 0.01-1.11 × 10^-3^ M in sweat, 0.05-5 × 10^-3^ M in tears, and 1.99-22.2 × 10^-3^ M in ISF) 13, 25. Therefore, an optimized design and analysis method for monitoring systems specialized for each body fluid method should be developed.

The sampling method and physicochemical properties of each body fluid are also important considerations. For example, ISF fills the spaces between most of the body's cells and provides a significant portion of the body's liquid environment 13. ISF has potential for medical diagnosis because it is very similar to whole blood plasma in terms of composition. Subcutaneous injection of a needle is required to monitor ISF, which can be inconvenient for prospective users 13. Saliva is a complex mixture of 99.5% water and 0.5% electrolytes (glycoproteins, lipase, mucin, amylase, glucose, and antimicrobial enzymes) 26. Analytes can be easily collected by spitting, but saliva contains many impurities, making it difficult to isolate the intrinsic glucose in the fluid. In tears, they can be excreted from the body as an extracellular fluid containing mucin, lipids, glucose, water, lysozyme, lactoferrin, lipocalin, lacritin, urea, sodium, immunoglobulins, and potassium 25, 26. Tear glucose concentration has been shown to be highly correlated with blood glucose, with relatively little interference by impurities. However, biosensors require high sensitivity, selectivity, and low limit of detection (LOD) to monitor tear glucose concentration. Lastly, sweat is composed of water, ammonia, urea, salts, and glucose. The glucose level in sweat shows a high correlation with that in whole blood 27. Sweat is easily accessible, but it is complicated by impurities and other components and requires exercise for collection.

All of these approaches using body fluids for blood glucose monitoring have unresolved hurdles as their respective fluid-based glucose measurements have not been strongly correlated with plasma glucose concentration or clinical evidence. However, painless and noninvasive methods using each body fluid-based glucose measurement are promising as they allow faster and more convenient monitoring of glucose concentrations in diabetes patients. Therefore, for the finding high correlation and clinical evidence, a system capable of requiring more accurate measurement is needed, and many clinical trials are required.

## 3. Glucose monitoring techniques

**Figure 2:**
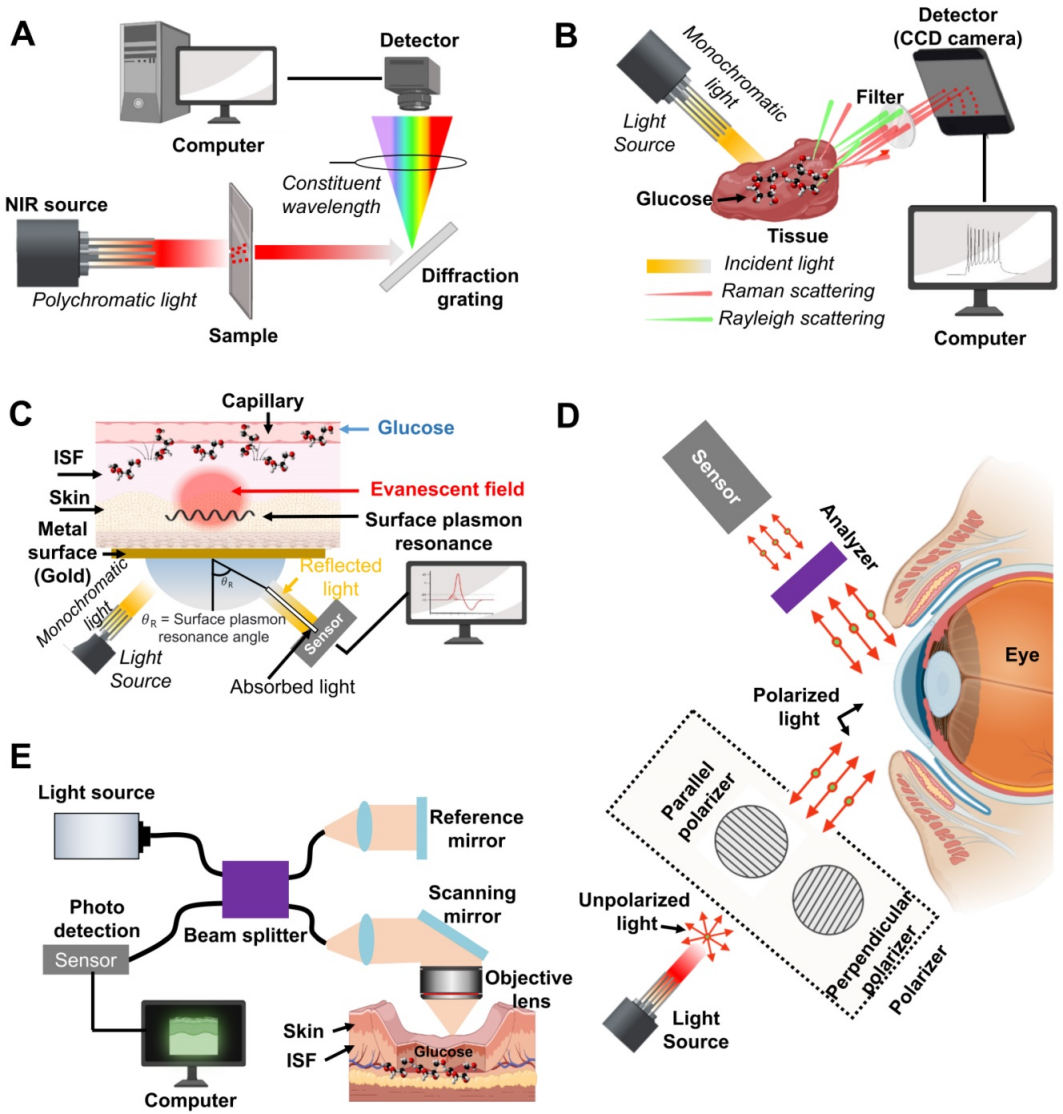
** An illustration of the principle for glucose detection using optical monitoring methods.** Principles of (**A**) NIR spectroscopy, (**B**) Raman spectroscopy, (**C**) Surface plasmon resonance (**D**) Optical polarimetry, and (**E**) OCT scanning. Reproduced with permission 43. Copyright 2019, MDPI

**Figure 3:**
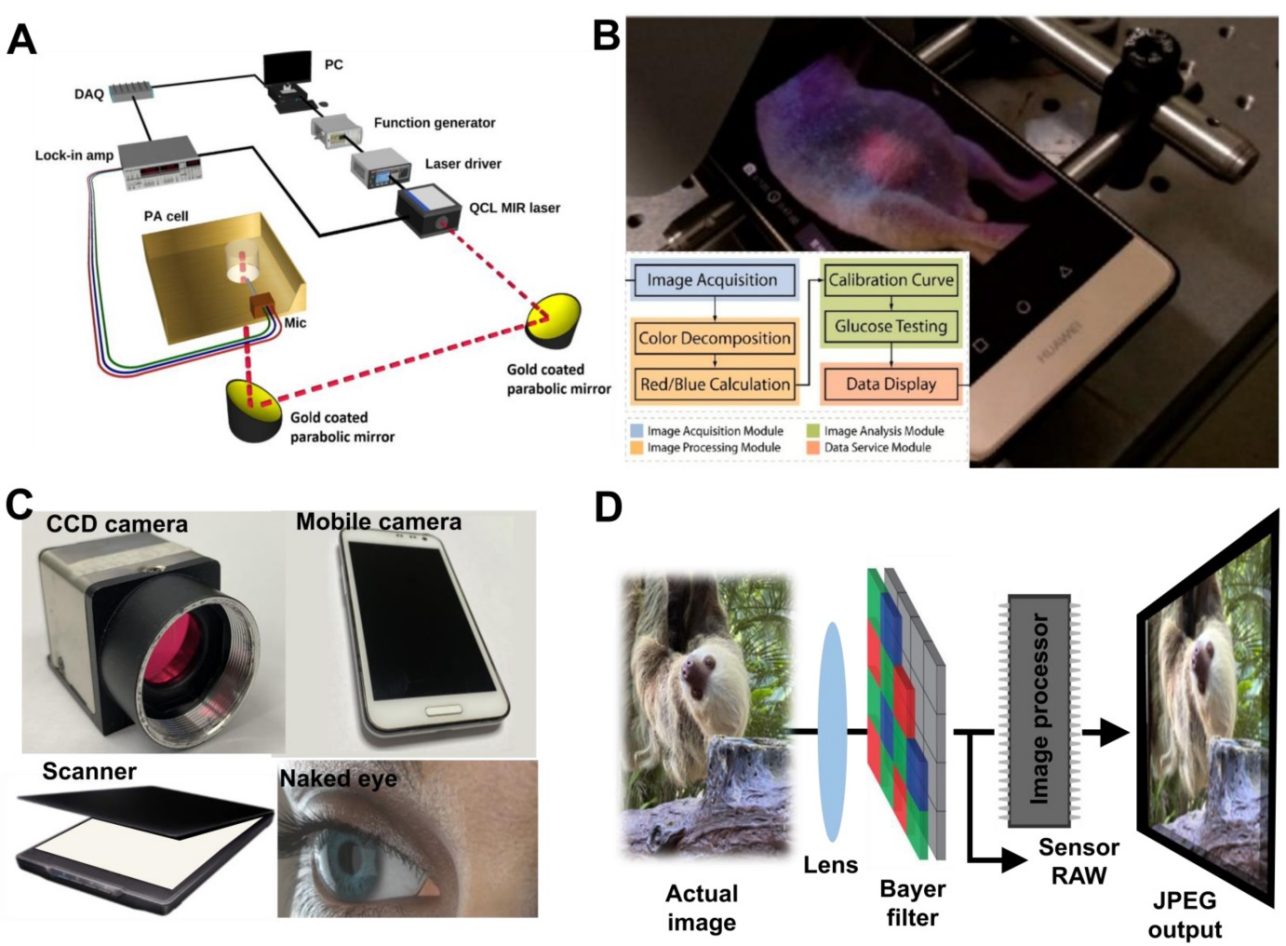
** An illustration of the principle for glucose detection with different types of monitoring methods.** Working principles of (**A**) photoacoustic spectroscopy for glucose monitoring. Adapted with permission from 53. Copyright 2017, MDPI. (**B**) optical transducer. Adapted with permission from 103. Copyright 2018, American Chemical Society. (**C**) Different devices used for image acquisition of the colorimetric reaction: (left-top) sCMOS, CCD-based digital camera (right-top), smartphone (left-bottom), image scanner (right-bottom), and the naked eye. (**D**) The working principle of the image sensor from the colorimetric reaction.

Developing a practical glucose biosensor with high reliability and sensitivity must be a top consideration for the biosensor industry. Various noninvasive glucose biosensors have been developed including optical, electrochemical, transdermal, colorimetric assay, luminescent detection, magnetic signal detection, and microwave approaches 28. **Table 2** shows the advantages and disadvantages of LOD, nanomaterials, and techniques for glucose detection with different methods and the current research status using body fluids. Optical methods of near-infrared reflectance spectroscopy (NIRS) 29, 30, surface plasmon resonance interferometry 31 photoacoustic spectroscopy 32, polarized optical rotation 33, Raman spectroscopy 34, fluorescence 35 and optical coherence tomography (OCT) 36 are illustrated in **Figure 2, 3**.

Optical methods measure the glucose concentration from body fluids and indirectly calculate the blood glucose concentration using correlations between glucose concentrations in whole blood and body fluids.

### 3.1. Near-infrared reflectance spectroscopy (NIRS) and Raman spectroscopy

Arnold *et al*. reported noninvasive glucose monitoring in diabetic patients utilizing the visible and near-infrared (NIR) spectral regions 37, 38. A spectrophotometer can measure reflected/transmitted light intensities as a function of concentration, absorption coefficient, and sample thickness as well as evaluate the effect of scattered light in body fluids (**Figure 2A**). However, the internal structure of human tissues is complex, and spectral information of numerous materials or substances can interfere with the accuracy of the results. Therefore, NIRS methods require processing algorithms to extract the glucose concentration from multiple parameters such as refractive index, angle information, and information of the illumination light source. To improve this issues, deep neural network (DNN) model and efficient regression models has been designed for prediction of serum glucose concentration from healthy subjects, prediabetic, and diabetic condition 39. These DNN make more high accuracy serum glucose prediction in comparison with other NIR measurement with detection range of 80 - 420 mg/dL. However, since the training data and validation sets were relatively small and it can only detect prediabetic and diabetic condition, it is necessary to collect more clinical sample for the training and validation purpose for the detection of hypoglycemic ranges.

Raman spectroscopy is a non-destructive chemical analysis technique with sharp spectral peaks that provide direct chemical variations of the glucose molecule.* Raman scattering* is an inelastic scattering involving the target molecule's vibrational energy changes from interaction with incident photons. This wavelength change, called the Raman shift, can represent the glucose difference between the initial and final vibration states (**Figure 2B**) 37. Raman spectroscopy is performed at low frequencies at the end of the NIR band and detects fundamental vibrations of atomic groups, leading to more accurate identification. However, compared with other optical methods, Raman spectroscopy suffers from weak Raman signals, extremely challenging calibration in turbid samples, and strong background noise of the surrounding environments. Thus, current efforts are underway to resolve these issues in a wide range of techniques including photon migration theory and multivariate calibration (MVC) analysis for tissue modulation 40. However, to extract meaningful information, chemometric algorithms which is mathematical, statistical, and computational methods are required for interpreting analytical data. For this aspect, recently the combination of machine learning and surface enhanced Raman Spectroscopy (SERS), and convolutional neural networks (CNN) composed hidden layers are introduced and enhanced the Raman scattering. In 2022, Wang *et al*. introduced the Gramian angular field (GAF-CNN) which can convert into blood glucose concentration (output) with 1-D Raman spectral data (input) for prediction of glucose concentration. They showed the high performance with error of prediction (REMSP = 0.065) and coefficient of predication (0.999). These algorithms might be good option to predict of glucose concentration with Raman signal 41, 42.

### 3.2. Fluorescence spectroscopy

Fluorescence spectroscopy uses specialized molecules called fluorophores that absorb the energy of an excitation photon at a specific wavelength and then emit fluorescence, causing a wavelength difference known as the Stroke's shift 43. In glucose detection, fluorophores like intermediate molecules can bind directly to glucose and alter fluorescence 43. Fluorescent glucose sensing molecules may be configured to increase or decrease fluorescence at baseline depending on ambient glucose concentration. A variety of glucose-sensing fluorophore, ranging from inorganic (*e.g.* synthetic carbon nanotube materials and quantum dots) to organic (*e.g.* enzyme, glucose binding proteins (GBPs), and boronic acid derivatives), have been introduced over the decade 44. Quantum dots (QDs), nanometer-sized semiconductor crystal, can be designed to fluoresce at any wavelength depending on the crystal size and material used. There are three types of organic molecules that generally recognize glucose: (1) enzymes, (2) GBP, and (3) boronic acid derivatives. It consists of a fluorescent glucose sensor that can be integrated with a fluorophore to reversibly bind glucose. These organic molecules have the advantage of being able to monitor a wide spectral range from ultraviolet (380 nm) to near infrared (880 nm) depending on the immobilized fluorophore. Hitomi *et al*. reported fluorescence resonant energy transfer (FRET) as shown in **Figure 2C**, which provides excellent sensitivity for detecting low glucose concentrations 45. Fluorescence biosensors have advantages of high specificity, sensitivity, and a wide detection range, allowing measurement of analyte concentration based on fluorescence intensity and decay time due to the characteristic emission spectra of specific fluorophores. Therefore, *in vitro* test projects and experiments commonly use fluorescence biosensors. However, fluorescence biosensors have limitations, including potential toxicity issues, susceptibility to interference due to pH changes and oxygen concentrations, short lifespan of the fluorophore, photostability issues, loss of recognition capability, and a low signal-to-noise ratio. In addition, conventional statistical algorithms are often limited by low accuracy under low illumination conditions, long computation times, and incorrect initial assumptions of decay parameter 46. Therefore, machine learning-based simple training architectures of artificial neural network (ANN) or convolutional neural network (CNN) have been employed to improve the visualization, less computational time, and detect low fluorescent signal. In 2022, Chen* et al.* used a Wasserstein GAN-based algorithm in which the generator (G) is trained to generate a high-photon-count fluorescence decay histogram using a low-photon-count input. The GAN training algorithm with rectified linear unit (ReLU) activation was up to 2,800 times faster than the gold standard estimation and showed more accurate analysis of low-photon-count histograms. Since these algorithms have not yet been applied to fluorescence-based glucose detection, better neural network architecture with appropriate activation functions will eventually improve the issues.

### 3.3. Optical polarimetry (OP)

As another optical method, Cameron *et al*. developed optical polarimetry (OP) to noninvasively measure blood glucose concentration 47. They used a polarized beam of light to illuminate a glucose solution and measured the rotation angle of the incident light's polarized plane (**Figure 2D**) because glucose is an optically active substance and can rotate the plane of the polarized beam 37. The polarization orientation plane will be changed by the deflection angle from the original incident direction depending on the glucose concentration. The total rotation is proportional to the optical path length, concentration of the analyte, temperature, and wavelength of the laser beam (400-780 nm). Optical polarimetry takes advantage of the easy miniaturization of optical components because it only uses molecules that can rotate the plane of polarized light 48. However, the relatively low measurement accuracy is challenging issues, mainly due to the presence of other active molecules, high degree of scattering in skin tissue, varying corneal birefringence, and high deflection angle. To improve these issues, a number of geometric and physical model have been proposed to explain physical process, overcoming is difficult because extracting the sample itself is untidy, rendering unpopular sampling methods 49.

### 3.4. Optical coherence tomography (OCT) and photoacoustic spectroscopy (PAS)

Optical coherence tomography (OCT) has been developed to measure glucose concentration more accurately for high-resolution imaging, providing depth-oriented tomography with two- or three-dimensional images using low-coherence interferometry (**Figure 2E**). OCT typically employs the NIR range of light and can be used to observe internal biological tissues at depths of 1-2 mm with a spatial resolution of 10-15 microns. OCT was proposed to detect blood glucose from specific skin or *in vitro* samples and has distinct advantages including continuously monitoring blood glucose concentration with a high signal-to-noise ratio, high resolution, and high noninvasive penetration depth. However, OCT is sensitive to motion artifacts such as skin temperature change, pH, and humidity, leading to low measurement accuracy. In addition, variation of the scattering coefficient by physiological compound is a key factor restricting its development. Therefore, a photoacoustic spectroscopy (PAS) technique was reported by Tanaka *et al*. 50. The basic concept of PAS is shown in **Figure 3A.** The technology of PAS uses ultrasound waves and short laser pulses with wavelengths that are absorbed to produce microscopic-scale localized heating. The localized heating causes a volumetric expansion of the medium, generating an ultrasound wave that can be detected by an acoustic biosensor to measure variations of blood glucose concentration. Even though it can improve the depth and increase detection reliability on the micrometer scale 51, and has low detection sensitivity in the range of physiological glucose concentrations, PAS of the detection sensitivity is still unsatisfactory for clinically approved glucose monitoring. In 2018, Sim *et al*. developed *in vivo* microscopic PAS for noninvasive glucose monitoring. They used two laser sources (glucose absorbing and insensitive region detection wavelengths) to improve the system's overall signal-to-noise ratio (SNR) 52. In 2022, Abdulrahman *et al.* introduced a photoacoustic spectroscopy using machine learning to detect glucose level in skin sample with 40, 200 dataset and enhancing the sensitivity ± 25 mg/dLdL 53. Even though this study required to demonstrate the feasibility of *in vivo*, measurement of glucose concentration can be further enhanced with different classification models.

### 3.5. Luminescent detection

Luminescent detection-based assays have emerged with high glucose detection sensitivity based on fluorescence intensity to determine glucose variations in biochemistry and immunoreaction applications. Fluorescence emission requires excitation energy, usually at a shorter wavelength (**Figure 3B**). Luminescent detection is performed to capture the fluorescent intensity from luminescent nanoparticles such as quantum dots (QDs) 54, dye-doped nanoparticles 55 and up-converting nanoparticles 56. Similar to colorimetric detection, luminescent detection can be carried out on a handheld device that is cost-effective and easy to manage compared to bench-top apparatuses. In 2008, Faulstich *et al*. reported a pocket-size device that contains an illumination source and filters. The end-user inserts the test strip, activates the excitation light source, and monitors the test result with their naked eye 57. In 2013, to obtain more quantitative results, Kozma *et al*. developed a handheld fluorescent microarray reader consisting of a filter, CCD camera, laser diode (λ=635 nm) with a collimator, and a prism. The total measurement time was less than 20 s 58. In 2017, Zhang *et al*. introduced a more rapid system with an analysis range of 1-100 ng/mL 59. Conventional imaging setup required high quality of sCMOS or CCD, however, with the rapid development of sensing capabilities, the smartphone can capture luminescence image or fluorescence signal with expansion of attachments and software applications. Nonetheless, luminescent detection requires sophisticated optical components to result in high sensitivity.

## 4. Colorimetric assay

Colorimetric detection utilizes specific indicators (nanozyme or nanomaterials) that change color when interacting with molecules of interest. The color information is recorded with a complementary metal-oxide-semiconductor (CMOS) or charge-coupled device (CCD) divided into two-dimensional grids, known as pixels. The sensor converts the photons into electrons in each pixel. The electrical signal is processed by an image processor to produce final images in JPEG, PNG, TIFF, and BMP formats, as illustrated in **Figure 3C- D**. Digital cameras, scanners, and smartphones are now widely used to measure color changes and have emerged as suitable alternatives for colorimetric analysis. Among them, current smartphones are prominent as they are equivalent to a microcomputer with high-capacity internal memories and are equipped with high-resolution cameras and wirelessly communicate with other devices 60. Significant advances in smartphones make them useful for colorimetric assays, including fluorometric or spectroscopy applications.

In the algorithms aspects, conventional colorimetric analysis is started to track the region of interest (ROI), extracting the true color in color space of RGB, CMYK, HSB/HSL, CIE XYZ, L*a*b*, and YUV models. Each acquired intensity was directly plotted with prepared glucose concentrations, generating a calibration curve. This curve fitting was used to estimate sample glucose concentrations. However, colorimetric analysis is highly affected by ambient light conditions and camera optics. To address this issue, advanced algorithms such as machine learning based deep neuronal networks were proposed in the quantitative glucose evaluation process with automated decision-making and self-learning from the data.

### 4.1. Color space

RGB, CMYK, HSB/HSL, CIE XYZ, CIELAB, and YUV models are commonly used color spaces. The advantages and disadvantages of the color spaces are compared in **Table 3**. Primary RGB (red, green, and blue) or CMYK (cyan, magenta, yellow, and black) are the most used color spaces. In the RGB color space, each color is assigned to orthogonal coordinate axes in 3D space. The RGB color space ranges from 0 to 255 (8-bit format) or 0 to 1 (fractional format). The RGB color space is commonly used in industry, such as the Bayer filter used in CMOS sensors and many digital products (*e.g.* smartphones, webcams, flatbed scanners, digital cameras). Utilization of nanozyme in colorimetric-based glucose biosensors can typically use the RGB color space due to its simplicity. In 2022, Firdaus *et al.* developed smartphone application called glucose analyzer and built the Android Studio platform (DIC-Smartphone), which can achieve a detection limit of 0.043 μM 61. Although these algorithms can precisely extract RGB color values for calculation of glucose concentration, the RGB color space sometime omits a much smaller number of colors, indicating the limitations of the perceptual space.

On the other hand, the CMYK color space, mainly used for color printing, can be applied to paper-based glucose detection. Each color creates a range of colors from 0 to 100%. All colors are created from combination of different CMYK amounts. In 2018, Wilson *et al.* introduced a paper-based microfluidic device for glucose analysis employing artificial neural networks. They used 4-channel CMYK color data to demonstrate the effectiveness of ANN fitting and classification algorithms 62. Typically, the RGB colors from the images recorded by the camera were converted to the CMYK color space using Photoshop (Adobe, Inc., USA). However, since RGB can produce much more vivid colors than CMYK, a lot of data can be lost during this conversion.

The International Commission on Illumination (ICI) established CIE XYZ in 1931, the first system for scientifically defining light or additive colors. It is widely accepted as an international standard method for defining color. In CIE XYZ, chromaticity is defined by X and Z, and luminance is defined by Y. The calculated CIE XYZ coordinates are not the same as the original RGB values. The CIE XYZ color space is not as easily represented as the RGB color cube, but it is very similar to the RGB color space with noticeable color distortion. Spectrophotometers and digital color analytical instruments with a CCD or sCMOS sensor typically use the CIE XYZ color space because they can provide reflected or transmitted light from samples. In 2021, Samira *et al.* introduced a smartphone-based colorimetric sensing system for the measurement of glucose concentration in urine samples. They mapped image color acquired in the CIE chromaticity space, compared them with the reference color, and increased sensitivity with commercial reference charts 63. However, it is not easy to compare two colors due to the non-uniform color distribution.

HSB, also known as HSV, represents three components: Hue, Saturation, and Brightness. The HSB color space is a kind of non-linear transformation of RGB defined as the simple addition or subtraction of RGB color. Hue is the color type and can be defined as the length of the illumination spectrum ranging from 0 to 360º (for example, the value of 0º is red and 45º is a shade of orange). Saturation is a color range of high intensity values from 0 to 100%. Here 0% means no color and 100% is the most intense color. Brightness is the visual perception derived by luminance ranging from 0 to 100%, where 0% is colorless (black) and 100% is intense color (white saturated color). The HSL (hue, saturation, lightness) space is similar to that of HSB or HSV. However, the main difference of HSL is that it is symmetric to light and dark. Therefore, HSL usually provides a more accurate color approximation than HSB or HSV. This color space can represent a single parameter (H), avoiding redundant color coordinate information when use in colorimetric-based biosensors. In 2020, Simon *et al.* developed colorimetric sensor for glucose monitoring using smartphone camera. They used HSL and RGB color space to directly compare the estimated performance and show better results in the HSL models 64. However, this is not the same as the CIE XYZ color space. Thus, it is also difficult to find significant differences between the two-color spaces 108.

The L*, a*, and b* (CIELAB) spaces are international standard for color measurements adopted by the CIE in 1976. CIELAB consists of a luminance or luminance component (L*) between 0 to 100. The parameter a* is the color change from red to green. The parameter b* is the color change from yellow to blue. Unlike CIE XYZ and HSL, the CIELAB space is perceptually uniform due to slight change in the chromaticity diagram that produce changes perceived by the human eye. Thus, the CIELAB space can extract color differences between two colors, which is relevant for colorimetric-based biosensors. In addition, another advantage of the CIELAB space is that it uses Euclidean distance to determine the amount of color difference between two colors, providing information about color and time function changes for different glucose concentrations based on colorimetric detection via continuous and monotonic color profiles. In 2021, Son* et al.* introduced a colorimetric biosensor using deep neural network. To address the existing perceptual non-uniformity, they used CIELAB color space and found highly perceptible spectra with CIELAB coordinate values 65. However, the major disadvantage is the requirement of further processing and the number of steps needed to obtain the results.

As the last color space, the YUV model also describes color. The parameter Y is the brightness (luma component) in the range of 0 ~ 100%, the parameter U is the blue luminance of the chrominance component, and the parameter V is the red luminance of the chrominance component. In 2022, Yang *et al.* developed a biosensor for colorimetric determination of uric acid. For the colorimetric detection, they used algorithm in the YUV color space 66, because the Y value showed good linear relationship in the range of UA concentration. Moreover, the YUV model is suitable for detecting moving objects because YUV simulates human perception of color more closely than does the primary RGB color space model.

### 4.2. Image processing software and analysis formats

Digital images can be obtained from various devices (*e.g.*, smartphones, webcams, flatbed scanners, digital cameras) with different image qualities. Therefore, to quantitatively monitor the colorimetric variation, the same devices collect images to minimize instrumental errors. Image processing and analysis methods are essential to adopt colorimetric-based biosensors to reduce instrumental error and increase repeatability across devices. **Table 4** summarizes colorimetric glucose detection in body fluids. Many acquisition devices are based on the color change used for glucose monitoring. The RGB, CMYK, HSV/HSL, CIE XYZ, CIELAB, and YUV color spaces are usually processed using Matlab, Adobe Photoshop, Image J, Image color Picker processing, and Photometrix software. Matlab, Image J, and Adobe Photoshop are widely used in processing images obtained by digital devices 67. Several analytical parameters can be monitored after capturing digital images to measure colorimetric variation. In this step, a histogram is acquired, and various parameters can be calculated to obtain colorimetric differences. In addition, the Lambert-Beer Law (-log (*I*/*I*_0_)) or RGB normalization is used to determine a quantitative correlation of analyte concentration 19, where *I*_0_ refers to the background signal intensity (Black), and *I* refers to the sample signal intensity monitored from chemical or enzymatic reactions 68. However, CIELAB, CIE XYZ, or HSV are unrelated to color intensity. Thus, the use of images analyzed by different processing programs has been used as a strategy to determine a variety of analytes present in different matrices.

The image save format also affects the image quality of the color value in colorimetric-based biosensors. The detailed image format characteristics are described in **Table 5.** JPEG, also called JPG, is widely used to measure colorimetric variation due to their smaller file size, leading to fast image processing. However, the algorithm compresses images when creating JPEG, resulting in quality loss. Therefore, JPEG can lose some information that is not recoverable. Although GIF images undergo a different type of image compression that reduces the file size, they only use 256 colors, leading to poor image quality. PNG and BMP images use different image compression strategies without any quality loss and can be extended up to 24-bit color. Therefore, the images can be saved and analyzed without degradation. In addition, raw TIFF and DNG files are much larger, which means they require more storage capacity and result in a longer transmission time for image processing. However, TIFF and DNG images offer exceptionally detailed and high-quality images. Thus, these formats allow the ability to identify slight differences of color in colorimetric-based biosensors. Recently, smartphones have emerged as attractive capture devices because other devices cannot independently handle the image information and must connect to a computer for colorimetric detection and measurement. Moreover, various smartphone applications exist that can be used to capture digital images, controlling the macro, focal length, brightness, and exposure time. Recent smartphones produce raw image files of DNG, offering numerous advantages for colorimetric-based biosensors.

In general, colorimetric-based biosensors enable quantitative evaluation of glucose concentration with simple image processing without much consideration of errors resulting from illumination from external light sources. However, a proper lighting position, angle, and power control are essential to increase precision and accuracy. Moreover, the focal length and exposure time difference are essential factors because they can affect the sensor's color recognition (CCD or CMOS) 69. Thus, measuring accurate color values is not trivial and requires specific control. Methods to reduce experimental error in other research fields can be used, such as normalization, training-based color reconstruction, color checker-based digital image reconstruction, auto-tracking, and real-time monitoring algorithms.

### 4.3. Colorimetric-based glucose detection with nanozyme

**Figure 4:**
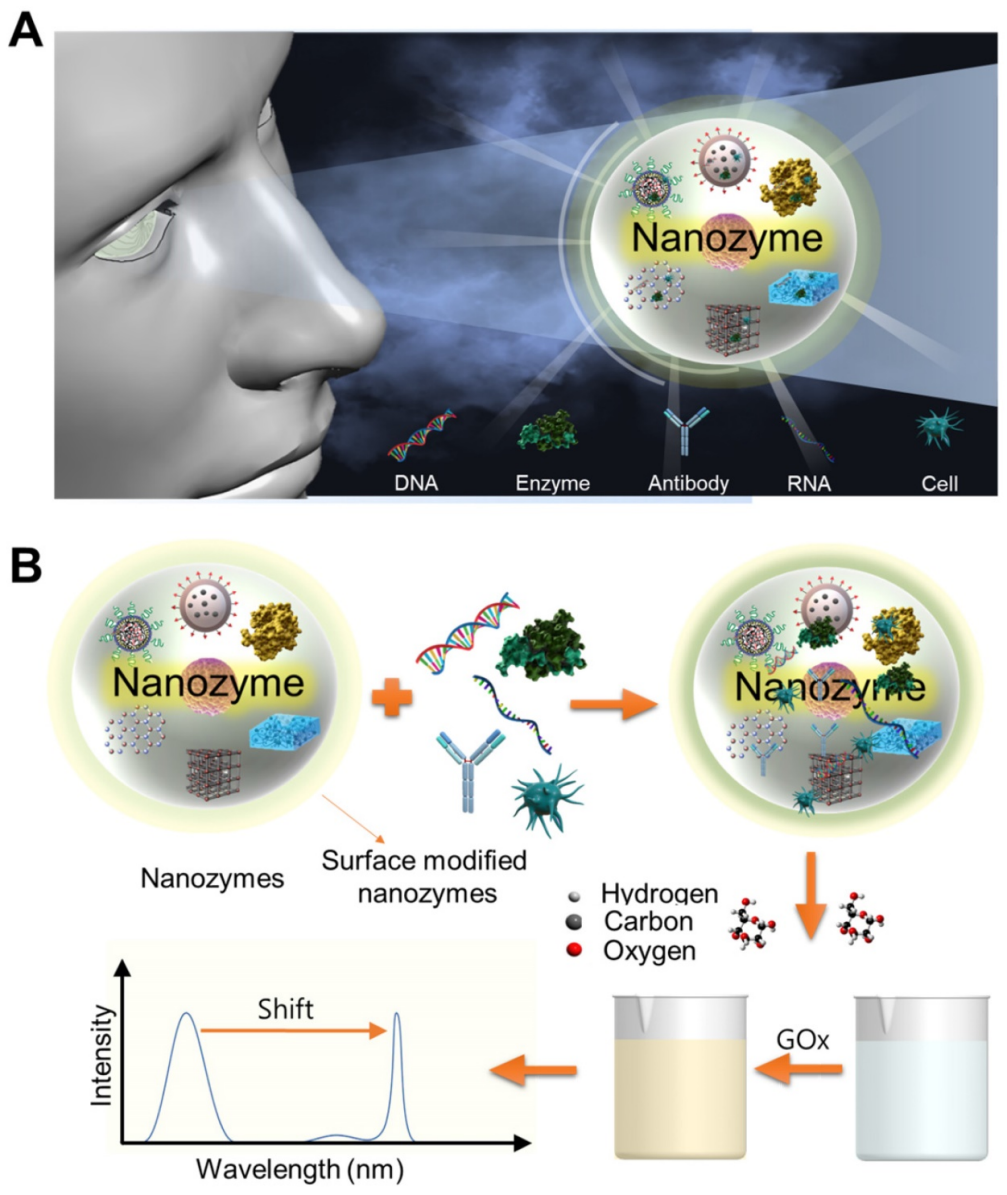
** Nanozyme-based colorimetric biosensor with color classification.** (**A**) Nanozyme based colorimetric detection with naked eye. (**B**) An illustration of nanozymes through assembly with peroxidase mimics.

Nanomaterials of natural enzymes have attracted enormous attention due to their unique characteristics compared to their molecular and bulk counterparts 70. However, with the exception of some catalytic RNA molecules, all natural enzymes have some intrinsic disadvantages. Some of the fundamental limitations of natural enzymes include their low stability, storage difficulty, high cost, tedious purification processes, limited application conditions, and specific operating conditions (i.e., narrow substrate, temperature, and pH ranges). For example, degradation upon exposure to various environmental conditions is a risk factor, and natural enzymes are particularly susceptible to digestion by proteases. They also require time-consuming preparation and purification processes, relatively high costs, and specific storage conditions. Therefore, nanomaterial-based artificial mimetic enzymes are receiving considerable attention. Recently, nanomaterials with 'enzyme-like' activity that mimic traditional biological catalysts such as catalase, oxidase, and peroxidase have attracted interest for potential applications as artificial enzymes. Several engineered nanoparticles (NPs), called nanozymes, have been used as active substances in bioassay, biosensor, and biomedical fields 71, 72. These NPs include nanomaterials such as simple metal and metal oxide nanoparticles, metal‐organic frameworks (MOF), metal nanoclusters, nanotubes, nanowires, carbon dots as well as quantum dots. These versatile nanomaterials can exhibit enzyme-like catalytic capabilities while overcoming many of the stability limitations and effective range associated with natural enzymes. In addition, the applications of hybrid, synthesis, and stimulus-responsive advanced nanozymes could revolutionize current practices in life sciences and biosensor applications. According to the activity they exhibit, nanozymes are classified into two large families: the oxidoreductase family and the hydrolase family. Nanozymes that are involved in redox catalysis and function similarly to oxidase, peroxidase, catalase, superoxide dismutase, or nitrate reductase are classified in the oxidoreductase family. As shown in **Figure 4**, by modifying the surface of iron oxide nanozyme or glucose oxidase or configuring it in a hybrid form, it is possible to construct a nanozyme capable of performing the function of an oxidase. In this assembly, oxidase activity is crucial as it provides a peroxidase-like nanozyme with hydrogen peroxide to induce a color change or emit light in colorimetric or fluorescent biosensors. These synergistic characteristics have led to ultra-sensitive platforms and high performance, including colorimetric, fluorometric, chemiluminescent, surface-enhanced Raman scattering, and electrochemical biosensors.

The most widely studied nanozymes in these biosensing systems are summarized in** Table 6,** including metal nanoparticles (NPs), metal oxide NPs, and carbon-based nanomaterials. Various nanomaterials, such as Fe_3_O_4_ NPs, Co_3_O_4_ NPs, carbon nanotubes, and graphene oxide, have peroxidase mimic activity 73, 74. In addition, CNPs have been demonstrated to have peroxidase-like activity, which can be applied to design corresponding colorimetric sensing systems 75. The peroxidase-like activity of iron oxide nanocomposites has been widely used for glucose detection. As peroxidase-mimicking nanozymes can oxidize chromogenic substrates (*e.g.* TMB, ABTS, and OPD) and produce color in the presence of H_2_O_2_, they can directly detect H_2_O_2_ or other H_2_O_2_-generating substrates (*e.g.* glucose). In all cases, these materials were combined with GOx, and the synergistic effect of these two enzymes is a key factor in achieving high sensitivity and superior analytical performance in biomolecular detection. The superior activity of these nanozymes facilitated colorimetric assays of H_2_O_2_, glucose, and sarcosine. In addition, it is possible to increase the effective catalytic surface area by introducing pores into iron oxide nanoparticles and to increase glucose detection sensitivity by exposing metal ions to the surface.

Recently, supramolecular peptide nanomaterials are attracting attention as candidates for constructing organic nanozymes because peptides and enzymes are composed of amino acids as basic units 76. These organic nanozymes can be rationally developed through self-assembly of peptides containing key amino acid sequences that participate in the formation of catalytically active sites. Amphiphilic amino acids can serve as building blocks for the supramolecular construction of nanoassemblies with the help of cofactors such as metal ions, and offer the possibility to fabricate nanozymes with minimal biological building blocks 77. Several organic nanozymes studied so far are inspired by the supramolecular structure and redox principle of horseradish peroxidase (HRP), a typical natural metalloenzyme that catalyzes oxidative substrates by H_2_O_2_. In this regard, histidine in native HRP plays an important role in participating in oxidation reactions and providing binding sites for coordination interactions with iron in heme. Therefore, inspired by the supramolecular structure of HRP, Geng *et al.* reported an organic nanozyme by co-assembly of an amphiphilic amino acid (Fmoc-histidine, FH) and a heme derivative (hemin) 78. The FH/hemin assembly showed flexible nanostructures and morphologies by tuning the molar ratio between FH hemins and optimizing catalytic activity to establish a sensing platform for rapid and sensitive glucose detection.

Metal-organic frameworks (MOFs) are emerging as important candidates in the field of glucose sensing. MOFs are a class of materials composed of organic ligands and metal nodes. MOF nanozymes exhibit additional properties due to their diverse structures and functions compared to nanozymes based on noble metals, carbon materials, or transition metal compounds. Strong coordination interactions between metal ions and organic ligands allow the formation of unique framework structures with multimodal properties; (1) the porous structure of the MOF provides abundant surfaces and channels for rapid mass transfer; (2) the specific pore size of the MOF is conducive to the loading, adsorption and separation of the target; (3) the presence of metal nodes in MOFs contributes to possible active sites for catalysis; (4) The organic ligands of MOFs provide attractive electrical, optical and thermal properties and abundant functional groups for chemical modification 79. Mechanically, the enzyme-like catalytic ability of MOFs can be attributed to two aspects: First, MOFs containing metal nodes such as Fe, Ce, Cu, Co or Ni can provide enzyme-mimicking catalytic activity due to the presence of these metal redox pairs. On the other side, the organic ligands of MOFs act as electron mediators, accepting electrons from a substrate and then donating electrons to other substrates, facilitating reactions similar to natural enzymes. Recently, several MOFs have been introduced as promising nanozymes with peroxidase-mimicking activity for glucose detection. In the study of Lin *et al.*, terephthalic acid (TA) was used as a crosslinking ligand for MIL-53(Fe), which was applied as a fluorescent probe for hydroxyl radicals 80. Fluorescent products were generated under the catalysis of H_2_O_2_ by MIL-53(Fe) MOF-based nanozymes, and the fluorescence intensity of the sensing system was related to H_2_O_2_ and glucose concentrations. Shahrokhian *et al.* reported an *in situ* strategy for the direct growth of Co_3_(BTC)_2_ MOFs on free carbon electrodes 81. Electrodes designed for glucose concentration detection exhibit two linear ranges: 1 µM to 0.33 mM and 0.33 to 1.38 mM, with sensitivities of 1792 and 1002 µAm/M/cm^2^, respectively. In the context of colorimetric glucose sensing, glucose oxidase@Cu-hemin metal-organic frameworks (GOD@Cu-hemin MOFs) with ball-flower structures as bi-enzyme catalysts for glucose detection have been reported by Lin *et al.* 82. The absorption intensity of oxTMB increases linearly with increasing glucose concentration from 0.01 to 1.0 mM, with a detection limit of 2.8 μM, which is claimed to be reasonably designed for colorimetric glucose sensors. Another example of MOF for glucose detection was designed to fabricate a nanoassembly by binding an amphiphilic amino acid, a histidine derivative, to a heme derivative containing an iron ion at the center 83. The iron ion of the heme derivative and the side chain of the histidine derivative interact non-covalently and exhibit peroxidase mimicking properties that can confer glucose sensing ability.

Although electrochemical-based biosensors utilizing nanozymes demonstrate highly selective and sensitive performance, they require sophisticated fabrication, storage capacity, electrodes, and chips for wireless communication. These drawbacks have led researchers to design glucose biosensors that do not require electrodes. It would be very useful if colorimetric biosensors could achieve the same performance as electrochemical biosensors. Therefore, nanozymes in colorimetric-based biosensors are important to achieve cost-effectiveness, high sensitivity, high selectivity, and stable biosensors for diagnosis and management of diabetic patients. Colorimetric glucose detection using nanozymes has the advantage of providing a rapid response (color change) to obtain visual observation (color camera and naked eye) 84. After the first results are obtained, the concentration and severity of the disease can be quantified using a color camera (CCD or CMOS) or other quantitative measurements to determine the management strategies and treatment options. Additionally, the digital cameras, scanners, and smartphones are now widely used to measure color changes and have emerged as suitable alternatives for colorimetric analysis. Among them, current smartphones are prominent as they are equivalent to a microcomputer with high-capacity internal memories and are equipped with high-resolution cameras and wirelessly communicate with other devices. Significant advances in smartphones make them useful for colorimetric assays, including fluorometric or spectroscopy applications. It might be overcome using smartphone-based glucose detection platforms for widespread use and better sensitivity for self-diagnosis and management of diabetic patients. Also, algorithm software, including machine learning-based detection and polynomial regression, can improve sensitive glucose detection. Therefore, colorimetric-based glucose detection using nanozymes is suitable for self-monitoring of glucose because of its rapid and cost-effectiveness glucose detection.

## 5. The current colorimetric analytical devices with nanozymes

### 5.1. Paper-based colorimetric glucose biosensors

**Figure 5:**
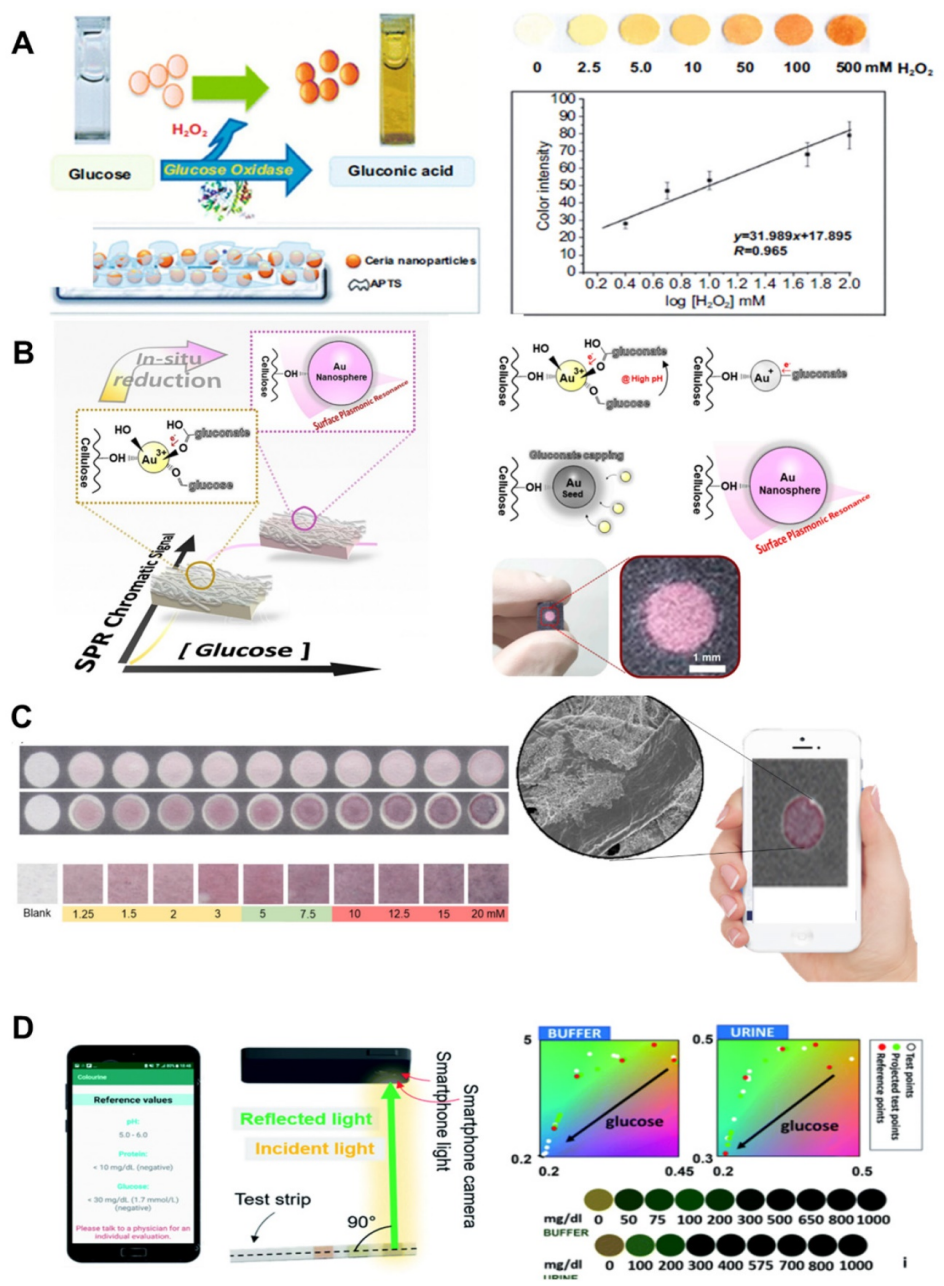
**A paper-based colorimetric glucose biosensor.** (**A**) Paper strip to detect H_2_O_2_ and glucose utilizing a cerium oxide (CeO_2_)-based bioactive biosensor. Adapted with permission from 86. Copyright 2011, American Chemical Society. (**B**) Chromatic characteristic of plasmonic paper with gold nanoparticles (AuNPs) formation in the CIE XYZ in 1931 color space. Adapted with permission from 87 Copyright 2020, MDPI. (**C**) Schematic illustration of the ATO-based paper biosensor as peroxidase mimics for colorimetric detection of glucose using smartphone read-out. Adapted with permission from 89, Copyright 2019, Springer. (**D**) User interface with smartphone readout of glucose urine tests using smartphone app. Adapted with permission from 63, Copyright 2021, Royal Society of Chemistry.

A paper-based colorimetric detection with nanozymes consists of the conjugation of sample analyte, detection, and signal amplification. Paper can serve as the base material for biosensing platform with a significantly lower manufacturing cost. This assay platform is promising for glucose monitoring. To this end, nanozymes enable low-cost glucose detection and have been used as high-sensitivity probes for detection and signal amplification 85. One of the important advantages of using nanozymes is that they can be easily synthesized without expensive chemical and sophisticated instrumentation, reducing the overall manufacturing cost. This allows the application of numerous metals, metal oxides, and MOF nanozymes for inexpensive colorimetric-based biosensors. Ornatrska *et al*. introduced the fabrication of a cerium oxide (CeO_2_)-based bioactive sensing paper strip to detect H_2_O_2_ and glucose concentrations (**Figure 5A**). With a reproducibility of 4.3%, this paper-based detection can be used for a minimum of 10 cycles without loss of activity, reducing costs in each experimental cycle 86. The basic concept of this study was to use simple electrostatic adsorption method using functionalization of CeO_2_ nanoparticles. Glucose oxidation produces higher concentration of H_2_O_2_, and the physicochemical properties of CeO_2_ change with oxidation state, resulting in colorimetric detection of glucose and H_2_O_2_. The acquired images were analyzed using Adobe Photoshop software, and the blue color intensity are mainly monitored because blue is the complementary color of yellow/orange. Reusability and the use of cost-effective materials are the main advantages of this study. Another advantage of nanozymes is their high thermal stability and mild storage conditions, which can reduce manufacturing costs. Although there are significant advantages in terms of cost-effectiveness from the materials, the acquired image system is bulky and inconvenient for the end-user.

In 2018, Tran *et al*. developed a nanocomposite using peroxidase mimicry to detect glucose in human urine using FEOOH and N-doped carbon nanosheets. They demonstrated Fe-CN nanocomposite stability for up to 90 days. Another excellent example of low-cost biosensor fabrication utilizing highly stable nanozymes was established by Kim *et al*. (**Figure 5B**) 87. Their work utilized plasmonic paper-based gold nanoparticle formation (AuNPs) and detected color change. An image of RGB values acquired from a scanner (1200 dpi) was converted into CIE XYZ in 1931 space. Glucose concentrations were estimated using exponential smoothing curve fitting. They showed a high linear correlation (R^2^ = 0.97) and similar sensitivity within low glucose concentrations. These studies demonstrate that nanozymes reduce manufacturing cost and provide much higher detection sensitivity compared to natural enzymes. However, despite the cost-effectiveness and facile synthesis of nanozymes, color change measurement requires expensive and bulky UV or visible spectrophotometer equipment, which can be overcome using smartphone-based glucose detection platforms.

Currently, smartphones have become an essential part of our lives and are being used for scientific purposes. Most smartphones have built-in sensors such as Bluetooth, HD (high definition) cameras, USB (universal serial bus) ports, thermometers, microphones, and gyroscopes. These features make smartphones an attractive platform for analytical devices in environmental monitoring and disease surveillance 88. Li *et al*. first reported Antimony-doped tin oxide nanoparticles (ATO NPs) loaded on a filter paper mimicking peroxidase-like activity, and combined them with a smartphone to an analyzer that detects H_2_O_2_ and glucose 89. This approach was used to determine glucose in aqueous samples. In addition, for the development of rapid, disposable, cost-effective manufacturing and inexpensive devices, Pinheiro *et al*. developed a colorimetric paper-based assay for determination of glucose concentration determination 90. They synthesized gold nanoparticles (AuNPs) by reducing the gold salt precursor to directly measure glucose using a smartphone camera in **Figure 5C**. Smartphone cameras utilizing nanozymes for colorimetric glucose detection show harness portability, high-speed processing, and high sensitivity. However, the development of digital systems that can interface with mobile sensors in an efficient manner remains a challenge because of the low sensitivity and optical noise of ambient light. In 2021, Balbach *et al*. reported a smartphone application that estimates colorimetric signals for glucose detection. As shown in **Figure 5D**, they put in a reference color chart and a CIE-RGB to HSV color space conversion to remove background noise provided by ambient lighting. These efforts have been devoted to the development of smartphone applications for colorimetric detection. Such a system would provide benefits to diabetics who need to constantly monitor their blood glucose levels on a daily basis.

### 5.2. Microfluidic paper-based device for glucose biosensors

**Figure 6:**
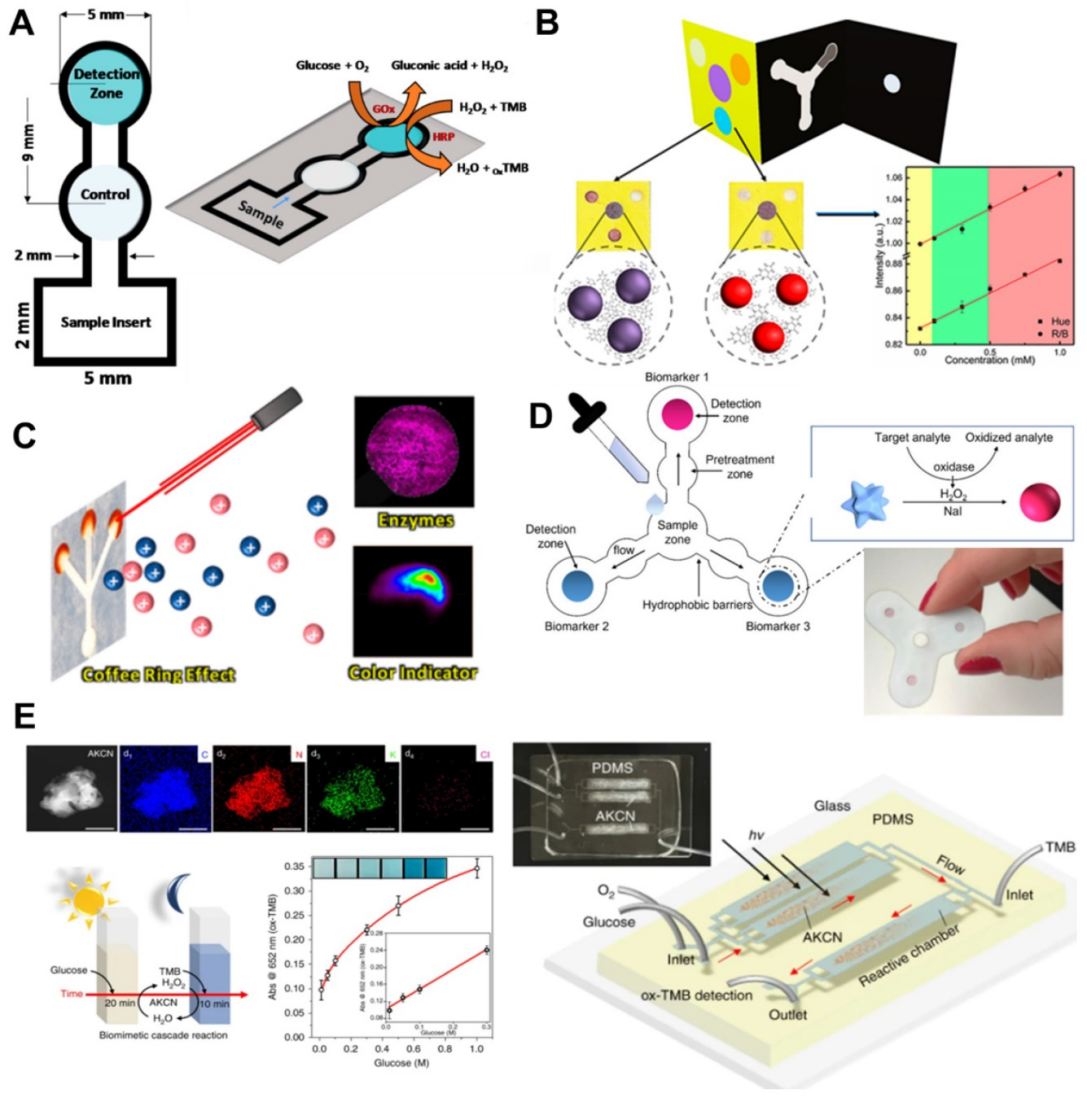
** The glucose biosensor of microfluidic paper-based devices (μPADs**). (**A**) The layout of µPAD assays of the enzymatic reaction for glucose detection. Adapted with permission from 127. Copyright 2017, MDPI. (**B**) Scheme of paper microfluidics and tailored AuNPs, colorimetric multiplex biomarker detection. Adapted with permission from 93. Copyright 2011, American Chemical Society. (**C**) Illustration of microfluidic paper-based analytical device for glucose colorimetric assay by mass spectrometry imaging. Adapted with permission from 128. Copyright 2018, American Chemical Society. (**D**) The schematic illustration of the paper-based multiplexed colorimetric device with microfluidic pattern for detection of salivary biomarkers. Adapted with permission from 126. Copyright 2021, MDPI. (**E**) Comparative enzymatic cascade reaction for glucose detection and scheme in a microfluidic device. Adapted with permission from 98, Copyright 2019, Nature Publishing.

The performance of microfluidic paper-based devices (μPADs) has been extensively investigated for monitoring glucose concentrations 91. μPAD is attractive for the following reasons: first, μPAD is ubiquitous and consists of very inexpensive materials. Second, μPAD is compatible with other chemical, biochemical, and medical applications. Third, μPAD uses capillary forces to transport liquids without external forces. Various two-dimensional (2D) and 3D microfluidic channels have been made on paper, which transport body fluids in pre-designed μPAD pathways, allowing quantitative detection of glucose concentrations 92. Coltro *et al*. reported a paper-based colorimetric biosensor for the measurement of surface acetic acid-to-chitosan-modified tear glucose 114. They measured glucose concentrations in human tears using TMB as a chromogenic reagent (**Figure 6A**). Images are recorded with office scanner at 600-dpi resolution and converted to RGB color space. Pinheiro *et al.* also presented the application of chemically produced and tailored AuNPs in μPADs for glucose sensing (**Figure 6B**). They also used commercial scanner to minimize the effect of ambient light conditions, and a AuNP-based plasmon for colorimetric transformation of paper substrate sand showed an LOD of 1.25 mM at a time of 2 min 93. Although μPAD is a cost-effective material and commercial scanners can also improve glucose detection sensitivity, the paper may fluoresce under prolonged illumination and interfere with true color detection. Pomili *et al.* introduced fully integrated all-in-one paper-based device to detect salivary glucose concentrations. They used colloidal 60-nm multibranched AuNP (MGNPs) and read the colorimetric response within 10 min with the naked eye or using a smartphone. Ortiz-Gómez *et al*. also reported a paper-based microfluidic colorimetric device for measuring glucose in urine and serum based on a Fe-MIL-_101_ metal-organic framework (MOF) 123. The assay was based on Fe-MIL-_101_ MOF to mimic horseradish peroxidase (HRP) immobilized on commercial cellulose paper. Their μPAD allowed for accurate measurement of glucose using a small sample volume (10 μL) with low LOD (2.5-10 μM/L). Images acquired with a smartphone can be processed on the same device as the developed iOS application without a separate attachment.

However, accurate measurement requires the system to set certain conditions, including shutter speed, aperture value, focal length, automatic white balance, and the same ambient lighting conditions. Also, the captured image is processed after saving the JPEG, where the raw color information can be lost. Therefore, in 2021, Mercan *et al.* reported a portable platform based on a color change in μPAD by implementing machine learning classifier with Linear Discriminant Analysis (LDA), Gradient Boosting Classifier (GBC), and Random Forest (RF) 94. They used different smartphones to train images captured in seven different lighting conditions. Among the tested and calibrated image sets, TMB (98.24%) with an LOD of 0.8 μM obtained the highest accuracy classification. The platform can automatically find ROI and minimize human error, contributing to user-friendly and accurate measurement of POC systems.

For improved sensitivity, Freitas *et al.* used mass spectrometry in combination with matrix-assisted laser desorption/ionization (MALDI) and desorption electrospray ionization (DESI) to monitor color gradient-based μPAD (**Figure 6C**). To understand assay performance such as reproducebility and sensitivity, they used a glucose enzyme assay using potassium iodide (KI) as a chromogen for generating color formation 93. Although MALDI and DESI imaging techniques have been successfully explored in enzymatic assays for glucose colorimetric detection, the above studies have reusability issues, information loss due to RGB color space conversion, and bulky imaging setups. From these points of view, inexpensive, simple and reliable self-monitoring image acquisition systems and algorithms are highly demanded for POC devices. For the development of POC devices, Wang *et al*. developed a smartphone-based spectrometer for colorimetric-based glucose biosensors containing aptamer-functionalized AuNP 95. The smartphone-based spectrometer is integrated with the grid substrate, and it uses the built-in camera and LED flash in the smartphone. They experimentally demonstrated the detection of glucose and human cardiac Troponin I (cTnl) with peptide-functionalized AuNP. A smartphone-based spectrometer with spectral numerical correction enables fast and sensitive real-time glucose monitoring with LOD from 0.2 to 0.47 mM. However, the integrated grating substrate is expensive and still requires additional devices and complex processing.

Meanwhile, to improve sensitivity, Darabdhara *et al*. fabricated paper strips to exploit the peroxidase and oxidase mimic activity 96. They prepared bimetallic Cu-Pd NPs to reduce graphitic carbon nitride (g-C_3_N_4_), graphene oxide (rGO) and MoS_2_ sheets with a size of less than 10 nm. They optimized the synthesis of Cu-Pd NPs with the desired shape, size, and oxidation state (**Figure 6D**). A designed biosensor strip of μPAD measured glucose in serum with a detection limit of 0.29 μM and a detection range from 0.2 to 50 μM 96. Tian *et al*. designed a 2D layer of PtS_2_ integrated with dopamine-functionalized hyaluronic acid (HA-DA) hydrogel microspheres for sensing H_2_O_2_ using a low-cost ultrasonication-assisted liquid exfoliation method 97. This biosensor has a greater color change than the PtS_2_ nanosheets by adding it directly to the glucose solution. Subsequently, a colorimetric biosensor based on PtS_2_ nanosheets and PtS_2_@HA-DA microspheres was developed to quantitatively determine glucose concentrations in buffer and human serum, respectively. The PtS_2_ nanosheet showed linearity in the dynamic range (0.5 to 150 μM) with a low LOD of 0.20 μM. Although a μPAD has been reported using this bifunctional oxidase-peroxide mimicking nanozyme and has provided a low-cost and simple platform for glucose detection, μPADs require reaction time to premix samples prior to the final reaction. From this point of view, inexpensive and metal-free bifunctional nanozymes using earth-abundant elements are highly desirable. Zhang *et al*. reported metal-free nanozymes of modified graphitic carbon nitride (g-C_3_N_4_: GCN) and demonstrated an enzymatic mimic role of this function 98. They demonstrated dual-functional enzyme-mimicking behaviors that combines the roles of oxidase (GOx) and peroxidase (HRP) using metal-free nanozymes based on modified graphitic carbon nitride (g-C_3_N_4_: GCN). In addition, they demonstrated bifunctional cascade catalysis in microfluidics for continuous colorimetric detection of glucose with an LOD of 0.8 μM within 30 s (**Figure 6E**). However, although this study showed a low LOD in microfluidic device that is sensitive enough for clinical glucose concentration, clinical validation and *in vivo* test are further required for practical application.

### 5.3. Colorimetric-based wearable glucose biosensors

With digitization of glucose monitoring, wearable systems are a convenient way for diabetes patients. Colorimetric-based wearable biosensors connected to digital cameras and smartphones can allow continuous monitoring by the end-user, while sample collection can be performed painlessly, an essential advantage for improving patient compliance 99. The development of wearable biosensors requires specific functions, such as soft, thin, and stretchable features 100. Therefore, the wearable biosensor withstands the physical burden and is in close contact with the body surface, making it convenient to wear and avoid physical perturbation due to skin contact. For example, if the material is hard, stabilization may occur during operation, which can affect measurement errors.

Different materials could alter the mechanical properties of the wearable glucose biosensors. Standard materials are textiles or fabrics, polymer composites, and papers that can be transformed into tattoos, patches, contact lenses, smartwatches, eyeglasses, and e-skin biosensors to act as sensors with integrated electronic devices 101.

**Figure 7:**
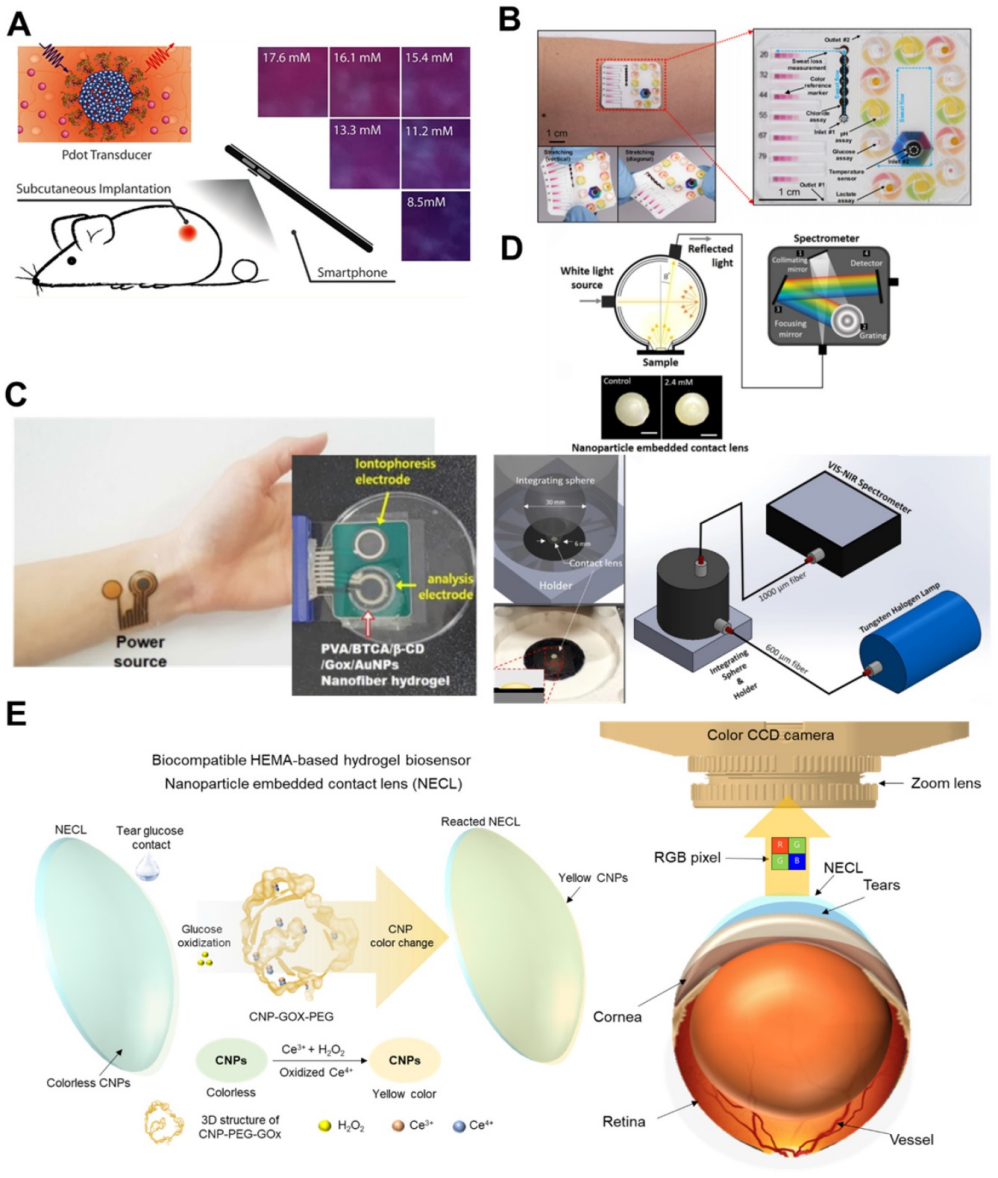
** Colorimetric-based wearable glucose biosensors.** (**A**) An implantable optical transducer using ultrasensitive luminescence signal detection for wireless glucose monitoring. Adapted with permission from 103. Copyright 2018, American Chemical Society. (**B**) A wearable microfluidic device using sweat on skin for colorimetric analysis. Adapted with permission from 21, Copyright 2019, ACS publication. (**C**) The images of wearable biosensor with glucose-responsive of the transparent nanofiber hydrogel patches. Adapted with permission from 129, Copyright 2020, Nature Publishing. (**D**) Schematic illustration of tear glucose measurement by the reflectance spectrum of a nanoparticle embedded contact lens (NECL) and configuration of the integrating sphere with external light source and spectrometer. Adapted with permission from 38, Copyright 2019, Nature Publishing. (**E**) Schematic illustration of colorimetric NECL and optical monitoring system. Adapted with permission from 24, Copyright 2019, ACS publication.

Microneedles (MNs) are introduced in implantable wearable devices to achieve instrument-free glucose detection and continuous blood glucose control. Ensuring the stability and accuracy of implantable wearable biosensors is very important for commercialization. Yang *et al*. reported a glucose biosensor without a noninvasive instrument 102. They designed a glucose-biosensing microneedle patch (GBMP) composed of GOx-conjugated MnO_2_/graphene oxide nanozymes (GOx-MnO_2_@GO) and swollen methacrylate gelatin (MeGel). GBMP is demonstrated by inserting into the skin to measure glucose concentration in ISF under hyperglycemic conditions to generate gluconic acid and H_2_O_2_ due to the enzymatic reaction of GOx and glucose in the body. Color changes are monitored with the naked eye or as RGB value with a smartphone camera. Color intensity is based on the quantitative analysis of glucose concentration and collects 5.1 ± 0.3 μL sweat from skin ISF within 3 min. Sun *et al.* also introduced an ultrasensitive optical transducer for wireless glucose monitoring with smartphone (**Figure 7A**). They developed oxygen-sensitive polymer dots (Pdots) and implanted subcutaneously on the dorsal side of mice for *in vivo* glucose monitoring 103. In order to clearly distinguish between normoglycemia and hyperglycemia, the image acquired with a smartphone was processed with an RGB model, and the contrast ratios of red and blue were compared. This design has the advantages of being easy to use, cost effective, high sensitivity, short measurement time, sustainability, and pain-free by detecting the color change of various samples. However, to increase accuracy by reducing measurement noise through advanced diagnostic algorithms such as machine learning and artificial intelligence, huge demand must be met.

In 2019, Choi *et al*. developed a sweat-based stretchable microfluidic device for the development of a patch types wearable device 21. It is the most advanced colorimetric-based biosensor platform for commercialization by optimizing microfluidic designs, chemistry and device layouts to enable precise evaluation. This platform is capable of measuring sweat, sweat rate, sweat temperature, pH, glucose concentration, chloride, and lactate in a physiologically relevant range (**Figure 7B**). To reduce errors in this work, they adopted a reference marker and measured glucose concentrations of ~0.1 μM. The integrated color reference markers can provide accurate colorimetric estimation of body fluids under various lighting conditions, and it is demonstrated at the different smartphone models in a remote setting. Koh *et al*. also reported that sweat is harvested and stored from human skin to measure glucose concentration, chloride, sweat rate, lactate, and pH levels 104. As shown in **Figure 7C**, tortuous channels within the microfluidic automatically collect sweat and image combined with a smartphone camera for RGB information. This allows the glucose concentration to be quantitatively determined from the RGB values. One distinct advantage of a sweat colorimetric-based wearable biosensor is that the wearer can interpret the qualitative analysis of the analytes in real-time without the need for an external device. However, wider use of the device requires the introduction of uncalibrated sensors associated with colorimetric glucose sensing systems. In addition, *in vivo* and clinical validation for meaningful medical applications is needed to monitor the association of sweat glucose concentration with blood readings.

Tear-based continuous enzyme sensing of a contact lens type, a wearable device, centers around glucose sensing, and current designs of intraocular nanozyme sensing have been incorporated into polymer matrix-forming contact lenses 105. In 2011, Yao *et al*. reported a contact lens sensing platform to measure glucose concentration by applying a GOD/titania sol-gel film immobilized glucose oxidase 106. They used Nafion® to decrease potential interference from the tear film and demonstrated real-time monitoring with a loop antenna, a wireless communication chip embedded in a polymeric contact lens. This kind of biosensor has fast response time (20 s) and dynamic range (0.1 - 0.6 mM). The 2014 Google glucose-sensing contact lens project was based on this work. In 2017, Kim *et al*. incorporated silver nanowires and graphene hybrid to improve the stretchability, conductivity, transparency, and contact lens-based biosensor 107. They designed a graphene/nanowire hybrid source-drain and graphene channel supported by biocompatible parylene substrate. This contact lens-based biosensor was *in vivo* demonstrated in rabbit and further *ex vivo* monitored glucose on a bovine eyeball. From this point of view, colorimetric detection using nanozyme is a promising method without requiring electrodes, a bulky system, energy storage capacity or detectors. However, the biosensors in this device are not generally biocompatible.

Moreddu *et al*. reported a paper-based microfluidic approach with contact lens 108. The glucose detection biosensor was a lab-made poly-HEMA contact lens combined with paper to detect multiple components, such as pH, ascorbic acid, glucose, proteins, and nitrite ions, in 2 μL of artificial tear in less than 35 s. In addition, they applied a smartphone application for glucose detection, converted to the CIE XYZ in 1931 chromaticity diagram to compare color changes, and used nearest-neighbor color search to return density values corresponding to the corrected color in the plot. These techniques can expand the number of calibration points and improve specificity by combining them within machine learning algorithm. Their paper-based microfluidic contact lens provided high sensitivity (3.9 nM/L) and LOD (1.1 mM/L) with reduced error under various lighting conditions. However, although this biosensor was not associated with toxicity issues, it is not evaluated in animal models *in vivo* and *in vitro* to understand body responses.

Recently, Park *et al*. developed a biocompatible biosensor of a nanoparticle-embedded contact lens (NECL) composed of complexes of cerium oxide nanoparticles (CNPs), glucose oxidase (GOx), and a polyethylene glycol (PEG) 20. After reacting with tear glucose and H_2_O_2_, NECLs are oxidized and changed into a yellowish color. A spectrometer was used for quantitative analysis of color change to measure glucose concentration using NECL. A detectable change in the reflection spectrum of NECL was found in relation glucose concentration (**Figure 7D**) 38. However, current spectrometers are expensive and require pre- and post-processing steps. Thus, they developed a simple RGB camera and smartphone-based optical monitoring system utilizing the NECL in animal models and clinical human tears 19. To reduce the environmental errors and enable continuous monitoring of the NECL in animal models, they developed an image processing algorithm that automatically optimizes the measurement accuracy even when images are obscured by motion artifacts (**Figure 7E**). This algorithm would be of great benefit to patients by enabling near real-time visualization and measurement of tear glucose concentration. In fact, they conducted blind evaluation of human tears provided by normal person and diabetic patients, and as a result, using the developed system, they were able to accurately discriminate between diabetic patients and normal person with a probability of over 90%. Furthermore, they evaluated the cytotoxicity of NECLs in human umbilical vein endothelial cells (HUVECs) and human corneal epithelial cells (HCECs). The cytotoxicity of NECLs was not noticeable, indicating that no problems occurred during contact lens fabrication.

## 6. Future direction

Recent research on nanozymes with enzyme-mimicking activity has overwhelmingly increased, expanding the scope of application of colorimetric-based glucose detection. Nanozymes integrated with colorimetric-based glucose biosensors have several advantages over natural enzymes as key functional components for analyte detection, including higher efficiency, greater versatility, lower cost, and higher stability. However, despite these significant advances, some research gaps and hurdles remain at these boundaries, and some work remains to realize the great potential of nanozymes in the development of colorimetric-based biosensors. First, although many nanomaterials have been used to mimic various natural enzymes in terms of material, oxidoreductase mimetics are mainly peroxidase mimics, because of their low-cost, easy storage, rapidity, and high sensitivity. Given the diversity of natural enzymes, additional efforts are needed to develop novel strategies for designing nanozyme with novel catalytic properties and machine learning based algorithms for quantitatively measure of glucose concentration. This increases the flexibility of nanozyme applications in biosensor development and provides affordability for detecting a wider range of analytes. Second, the sensitivity of colorimetric-based biosensors depends mainly on the catalytic efficiency of nanozymes. However, more attention should be paid to rational design of high-performance nanozymes to achieve the desired sensitivity-based colorimetric biosensors. For example, natural enzymes work together as enzyme clusters to provide high catalytic efficiencies within a confined environment for cascade reactions. For better sensitivity, nanozyme assembly is a good option, such as aggregation of other nanozymes or nanozymes with native enzymes. In addition, researchers have introduced several new materials with outstanding performance, such as the metal-doped composite materials or perovskite materials 14, 72, 74. Perovskite is a ceramic oxide with the molecular formula ABX_3_. The A is usually rare earth metal such as lanthanide and the B is a transition metal. Both A and B can be replaced by other metal ions of similar radius to form various compounds. There are studies that perovskite nanocomposites have high oxidase-like catalytic activity by a simple and accurate colorimetric detection method 109. Third, some studies have used other biosensing mechanisms instead of colorimetric signals for the growth of portable biosensors. However, the lack of portability and the existence of sophisticated and complex instruments limit the versatility of nanozymes using fluorescence or other sensing methods that rely on optical properties. Lastly, it has not been highly reported that studies on the toxicological mechanisms or potential toxicity of nanozymes identified so far. Since most of nanozymes are nanomaterials produced through metal-based bottom-up chemical synthesis, research on the toxicity of nanozymes is very important. For example, a previous study investigated the toxic effects of CeO_2_, a type of metal oxide constituting nanozymes 110. In that study, morphological-dependent cytotoxicity was confirmed by increased serum concentrations of lactate dehydrogenase (LDH) and tumor necrosis factor alpha (TNF-α) in rod-shaped CeO_2_ compared to cubic/octahedral CeO_2_. As shown in this example, a close toxicity analysis is required because there is a change in toxicity depending on the form, concentration, and degree of oxidation of the nanozyme even with the same component. The integration of nanozymes with wearable, implantable, and collectable biosensors requires careful control of the effective window to avoid potential toxicity, and systematic studies are required to evaluate the toxicity of nanozymes. Although previous reports have highlighted the therapeutic importance of several nanozymes in animal models, translating drugs into clinical applications remains a challenge 111. In addition, a thorough mechanism and understanding of the experimental phenomena under the practical application of nanozymes limits their rapid development for practical applications. The reason for this is that the relatively low catalytic activity and potential toxicity issues of nanozymes compared to natural enzymes make it very difficult to meet the practical application requirements of nanozymes. Therefore, this challenge remains a major hurdle to overcome in nanozyme applications in biosensing and will undoubtedly become an active area for future research. More research may be done in the future, especially to address issues above the processing step, simplify complex physical fabrication, reduce potential toxicity, and combine nanozyme monitoring systems with other technologies that continue to improve.

In terms of colorimetric analysis, naked eye detection is the simplest detection method. However, visual perception varies from person to person and depends on ambient conditions such as lighting. Therefore, in order to accurately and quantitatively measure the colorimetric change, a simple scanner or a digital camera that can accurately measure the colorimetric change is required. The advantage of digital devices is that no skilled manpower is required, high-resolution images can be generated, and color change can be quantitatively measured. Many colorimetric-based studies using smartphones to monitor glucose concentration have been heavily introduced due to their convenience and portability. However, there are several issues that need to be addressed using smartphones. First, images acquired from smartphone cameras are easily distorted and compressed, resulting in low correlation with real-world signals. The result is low repeatability, low accuracy, and low sensitivity in colorimetric-based glucose detection. These limitations are caused by ambient or environmental conditions, including varying lighting conditions, shooting distance, angle of the acquired image, and motion artifacts such as breathing and subtle movements. Second, different smartphone models have different RGB responses, so that different colors can be obtained from images processed by different smartphones. To reduce interference and increase accuracy, color correction methods using polynomial or linear regression algorithms using a color checker have been developed as basic information for color intensity 112. Accurate correction is performed by processing the acquired images through image normalization and white balance, brightness correction, saturation correction, and color transformation steps. Finally, accurate evaluation algorithms have been developed to improve the detection performance of *in vivo* models. Nowadays the application of machine learning in nanozyme will increase rapidly in the next few years, especially in material sciences and related fields as shown in **Table 7**. Because machine learning is great option, a subfield of artificial intelligence (AI) that uses statistics to improve accuracy techniques. It provides computer models with the ability to learn from a dataset, allowing models to perform specific tasks without explicit programming.

Artificial neural network (ANN) or convolutional neural network (CNN), and support vector machine (SVM) are very powerful ML model that can learn continuous functions using linear or non-linear systems with or without hidden layers. They show fast computation and easy implementation because they has fascinating features of learning 113. They only require input-output variables and identification of the relationship between processing parameters. Although they can adapt to real-time operations and calculate the fast result, they have little application in biosensors for colorimetric-based glucose detection. Additionally, the optical hyperspectral image (HSI) can provide more detailed color information, because it covers narrow spectral bands in the visible, NIR, and IR range instead of assigning primary colors (Red, Green, and Blue). Therefore, more detailed information can be achieved from colorimetric detection than the primary colors 114. However, spectrophotometers are bulky and expensive, making it challenging to apply glucose monitoring in various conditions. Recently low-cost hyperspectral imaging with smartphone techniques has been introduced 115. This is statistical learning-based image processing algorithms to be included in machine learning. They can convert RGB into hyperspectral image by training between actual measurements of spectral reflectance obtained from a hyperspectral camera or handheld spectrometer and RGB image captured by smartphone. Obviously, the reconstructed hyperspectral images computed on the smartphone allow for accurate glucose detection and are very useful. However, since the nanozyme-based colorimetric biosensor has not yet been integrated as a standard, additional work is required to apply clinical purpose, and it should be possible to calibrate the smartphone image obtained through it. Therefore, we argue that developing nanozymes with different enzymatic activities and developing various algorithms that can accurately detect color values ​​are excellent options for improving the accuracy of colorimetric-based glucose sensing.

## 7. Conclusion

The success of colorimetric-based biosensors utilizing nanozymes for glucose detection in diabetic patients requires high accuracy, user-friendly device portability, biocompatibility, and ease-of-use in image acquisition. Collectively, the challenges faced in design of colorimetric-based glucose biosensor applications are development of nanozymes. Rather than hindering future research, essential information collected from the current state-of-the-research is introduced along with detailed analyses to suggest future research directions.

## Figures and Tables

**Table 1:** Interstitial fluid (ISF)-based glucose monitoring comparison of body fluids for including whole blood, saliva, tears, and sweat

Body fluids	Biomarker	Typical concentration (mg/dL)	Typical concentration (mM)	Advantages	Disadvantages	Most studied Nano-materials	Ref
**Blood**	Glucose	80 - 120	0.33 - 6.66	Cost-effective, real-time monitoring	Invasive, highly uncomfortable for patients, infection risk	ZnO, metal NPs, metal oxide, CNTs, NiSe_2_-NS transition metal dichalcogenide, MOFs	130-132
**Urine**	Glucose	0 - 50	0 - 2.77	Noninvasive and painless, portable, rapid reproduction	Low accuracy, low glucose detection level, requires frequent calibration, susceptible to interference by bodily fluid	Metal NPs, CNTs, Pt, Ag@Au nanoprism-MOF	133, 134
**Saliva**	Glucose	1.5 - 4	0.08 - 0.22	Noninvasive and painless, safe for children and adults, easy sample collection, cost-effective	Low detection level, requires high sensitivity, inaccurate reading	Polymer, quantum dots, CNTs, Graphene, MOF-Encapsulated TiO_2_ Platform	135-137
**Sweat**	Glucose	1 - 4	0.06 - 0.22	Non or minimally invasive, sufficient quantities and rapid reproduction	Difficult sample collection, requires long calibration times, irritation and blistering of the skin, inaccurate readings, lag and inconsistent testing	Polymer,QDs, CNTs, Ni-based MOF	138, 139
**Interstitial** **fluid (ISF)**	Glucose	80 - 120	4.44 - 6.66	Painless, portable, quick results, long term use	Results and analysis influenced by multiple confounding factors, invasive, vulnerable to infection	Polymer, CNTs	140
**Tear**	Glucose	2.2 - 12.5	0.11 - 0.55	Highly accessible, less susceptibility to dilution, numerous testing methods, cost-effective	Lack of suitable power source for testing, requires low LOD, high sensitivity, high selectivity	Polymer, metal oxide NPs, CNTs, cerium nanoparticle (CNPs)	19

**Table 2:** Summary of the glucose detection methods, nanomaterials, limits of detection (LODs), advantages, and disadvantages

	Technique	Sample	Nano- materials	LOD	Advantages	Disadvantages	Ref
**Optical**	Infrared spectroscopy	Glucose solution	CuInS_2_ quantum dots (QDs) CuBDC	4.1 μM, 1.2 mM	Low scattering, low-cost materials, high penetration	Not portable, requires high hardware sensitivity, stability, and scanning pressure	29, 30, 141
	Raman spectroscopy	Saliva, urine	AuNPs onto Cu-tetra(4carboxyphenyl) porphyrin chloride (Fe(III))	3.9 μM	Sharpens spectra, less sensitive to temperature changes	Not portable, high cost, instability of laser wavelength, low SNR	34, 142
	Fluorescence spectroscopy	Glucose solution	Boronic acid Functionalized\CNPs, Carbon-based nanozyme CuAA	1.56 μM, 10 μM	Highly sensitive, less damage to the body	Not portable, high cost, scattering phenomena can affect accuracy	35, 143
	Surface plasmon resonance interferometry	Glucose solution	Gold in TiO_2_ coated in PCF, Gold nanoparticles	10 mg/dL, 25 μM	Rapid detection, real-time monitoring	Not portable, high cost, limited to high molecular weight biomolecules glucose, requires complex setup	31, 144
	Optical coherence tomography	Blood sample	Silica gold nanoshells	5.78 mM 0.015-0.045 mg/dL	High resolution, high penetration	Sensitive to individual movement, affected by temperature	36, 145
	Photoacoustic spectroscopy	Skin, Blood, glucose	AuNPs	0.035 - 0.098 μg/dL	High detection rate, high SNR	Sensitive to changes in temperature and pressure, vulnerable to motion artifacts	32, 146
	Optical Polarimetry	Tear, skin, glucose		125.4, 151.1 mg /dL	High resolution, easy to be miniaturized	Sensitive to temperature and motion changes, not portable, long lag time < 30 min	33
**Transdermal**	Impedance spectroscopy	Glucose solution, Skin	Gold nanoparticles	0.9 μM, 0.02-0.05 mg/dL	Differentiates between extracellular and intracellular fluids	Requires a long processing time	145, 147
	Reverse iontophoresis	Glucose solution	Gold nanoparticles PBNPs	0.01 mM, 0.85 μM	Biocompatibility, easy handling	Skin irritation, inaccurate and long-term measurement	38, 148
**Electrochemical**	Enzymatic detection of glucose	Tear, saliva, sweat, glucose solution	Gold nanoparticles SiO_2_, ZnO, Silver, WSNFs	0.1 μM, 3.7 μM 500 nM	Real-time monitoring, high detection range sensitivity, and low LOD	Requires electrodes, toxicity, vulnerable to temperature and motion change	149-151
	Amperometry	Glucose solution	Gold nanoparticlesPt	0.024 mM/L, 4 μM	Easily commercialized, high accuracy by multiple sensors	Sensor error from drift, calibration error and delays	152, 153
**Other**	Colorimetric assay	Tear, saliva, sweat	Cerium Nanoparticles	1.25 ng/mL 0.1 ng/mL	Rapid, widely used, reliable performance, portable and convenient, easy to manipulate, cost-effectiveness	Low accuracy, toxicity, limited by battery life and storage	58, 154
	Luminescent detection	Tear, saliva, sweat, H_2_O_2_	ZnO, Lanthanide-doped Nanoparticles and MOFs and CPs	5 ng/dL, 20 pg/mL 0.1 μM	High accuracy and sensitive, widely used	Not portable, need specific excitation source, high autofluorescence background, complicated operation	155, 156
	Magnetic signal detection	Tear, saliva, sweat	MoS_2_/Fe_3_O_4_ magnetic nanoparticles (MNPs)	100 nM, 0.5 ng/mL 8.6 nM	High accuracy and sensitivity, detect entire magnetic interference, portable, convenient, high accuracy	Requires an ultrasensitive magnetic sensor, vulnerable to external magnetic interference, high cost	157-159
	Microwave	Glucose solution	CuO nanoparticles	0.2 -10 μM, 36-454 mg/dL	High accuracy and sensitivity	High cost, toxicity, sensitive to temperature and motion artifacts	160, 161

**Table 3:** Comparison of color spaces for analysis of colorimetric detection

Color space	Color mixing	Primary parameters	Advantages	Disadvantages
**RGB**	Additive	Red, Green, Blue	Convenient for image acquisition and display	Non-uniform illumination, colors is not linear
**HSV/HIS**	Additive	Hue, Saturation, Value Hue, Saturation, Intensity	Based on human color perception; robust before non-uniform illumination, the chromaticity is decoupled from the intensity	Non-removable singularities
**CIE** L* a* b*, L* u* v*	Additive	L: Luminance, a: red to green b: blue to yellow u: Saturationv: Hue angle	Efficient in measuring small color differences, chromaticity is decoupled from the intensity	Singularity problems, nonlinear transformation
**CMYK**	Subtractive	Cyan, Magenta, Yellow, and Black	Commonly used for production printer color	Since it is a subtractive model, the components are pigments or inks
**YUV, YIQ**	Additive	Y (luminance), U (blue chroma), V (red chroma) I (rotated from U), Q (rotated from V)	Efficient coding color information for TV signal	The color range is restricted, difficult to recreate image display

**Table 4:** Summary of color spaces, image acquisition, and processing for colorimetric glucose analysis using various body fluids

Color space	Sample	Acquisition device	Acquisition format	Image Processing Software	Ref
**HSV**	Serum, urine	Canon Power shot S5 IS digital camera, iPhone 4.0	JPEG	Image J, Objective C	162
**SRGB**	Glucose solutions	Smartphone	JPEG	Image J, Gray color value filter paper	89
**SRGB**	Urine	Smartphone	JPEG	Android apps	61
**SRGB**	Glucose solutions, human serum	Smartphone	JPEG	Color picker, application n-pads	163
**SRGB**	Glucose solutions	iPod Touch	JPEG	Image J	164
**CIELAB**	Artificial sweat, human sweat	Smartphone	JPEG	Image J	124
**CMYK**	Glucose solution	Epson Perfection V600 scanner	JPEG	MATLAB image processing, toolbox, adobe photoshop, Photoshop CS2	62
**SRGB**	Artificial sweat, human sweat	Smartphone	JPEG	Smartphone based software	89
**SRGB**	Artificial sweat, human sweat	Smartphone	JPEG	Color grab application	165
**CIE-RGB-to-HSV**	Human urine	Galaxy A20e	JPEG	C++, Java, API 28 in Android Studio 4.0	63
**SRGB**	Glucose solutions, human serum, tears	iPhone 6	JPEG	Image J, Gray value filter paper	166
**RGB, HSL**	Glucose solution	Smartphone	JPEG	Android app	64
**SRGB**	Glucose solutions, artificial urine	Smartphone	JPEG	Urine analysis and android application	167
**SRGB**	Glucose solutions, artificial saliva	iPhone 7	JPEG	MATLAB	168
**SRGB**	Blood glucose	iPhone 5s	JPEG	Color assist application	169
**SRGB, RGB**	Whole blood Glucose solutions	Sony DSC-HX300, digital camera, Galaxy S5, Tab A, Moto G4	JPEG	Image J, Avidemux 2.6, Python, Open CV, Android studio	170
**SRGB**	Glucose solutions	Xiaomi MI 2SC	JPEG	Image J	171
**SRGB, RGB**	Glucose solutions blood glucose	Scanner (Epson perfection V700), LG Optimus Vu	JPEG	Image J	172
**HSV**	Glucose solutions, real samples	Smartphone	JPEG	Color Lab application	173
**SRGB**	Glucose solutions, human serum	LG Optimus L5 II	JPEG	Image J	174
**CIE LAB**	Glucose solution	Reflectance spectra	-	MATLAB,	65
**SRGB**	Glucose solutions	LG G2	JPEG	Image J, Microsoft, PowerPoint	175
**SRGB**	Glucose solutions	Smartphone	JPEG	Image J	176
**SRGB**	Glucose solutions, human serum	iPhone 6	JPEG	Image J, Adobe Photoshop, Gray value-μpad	177
**SRGB, RGB**	Glucose solutions human serum	I8000U, CCD (HDF70-A)	JPEG	Adobe Photoshop CS4	178
**CIELAB, SRGB**	Glucose solutions real samples	iPhone 5S, Samsung J5, Scanner (Canon MF 4780dn)	JPEG	C-Measure Lite, Color Grab, Digital Color meter	179
**SRGB**	Glucose solutions	HTC sensation XE, iPhone 5s, Nokia Lumia 920	JPEG	Cell phone spectrometer application	95
**SRGB**	Glucose solutions, urine	iPhone 4, Galaxy SII, MEIZU MX2	JPEG	Cam card, adobe photoshop	180
**SRGB, YUV**	Uric acid, human plasma	Smartphone, Microplate reader	JPEG	Color software	66
**SRGB**	Serum samples	iPhone 4	JPEG	ColorAssist application, RGB colors-commercial test slide	181
**SRGB**	Uric acid	Smartphone	JPEG	Image J, Android studio app	182
**SRGB**	Serum samples	Smartphone	JPEG	Image J-μPad	183
**SRGB**	Artificial urine	iPhone 5, Galaxy 5	JPEG	RGB colors-colorimetric urine test strips	184
**SRGB**	Glucose solutions	Smartphone	JPEG	Color detector application	185
**RGB, HSV**	Glucose solutions Human serum	Smartphone	JPEG	HSV application	186
**SRGB**	Human tears, glucose solutions	Smartphone	JPEG	MATLAB, ImageJ	20
**RGB**	Human tears, glucose solutions	Color CCD	TIFF	MATLAB, ImageJ	19
**SRGB**	Urine glucose	MIX6X Xiaomi	JPEG	Color Picker 1.5.2	103

**Table 5:** The characteristics of image formats for colorimetric glucose analysis and their advantages and disadvantages

Image Format	Available colors	Compression	File size	Advantages	Disadvantages
**JPEG (.jpg)**	16.7 million	Lossy	Small (<1MB)	Small image size, fast processing, widely used in the digital image	Loss some of the data files and not recoverable, lower image quality
**GIF (.gif)**	256	Lossless	Small (<1MB)	Suitable for animation as it enables transparency, good internet browser support	Few colors (256), low level of transparency support
**PNG (.png)**	16.1 milion + transparency	Lossless	Large (<3MB)	Extendable up to 24-bit color, can adjust color when displayed on different monitors	Lossless, large file size
**TIFF (.tif)**	Variable	Variable	Large (<3MB)	No compression, supported by image manipulation application, high image quality	Large file size, requires more storage data and long transmission time
**DNG (.dng)** **Raw**	Billions	No	Very large (<10MB)	Smartphone-based generation, 8 and 10 raw image files, high image quality	Large image file size, requires more storage data and long transmission time
**BMP**	Variable	Lossless	Large (<3MB)	Very easy to create, Simple to output	Does not allow image compression, low image quality

**Table 6:** Summary of nanozyme-based glucose detection.

Principle	Nano materials	Biological sample	Enzyme	LOD [uM]	Substrate	Optimum pH	Response time (min)	Ref
Colorimetry	Fe_3_O_4_-Au @ mesoporous SiO_2_ microspheres	Glucose solution	GOx	0.5	TMB	4.0	10	187
Colorimetry	V_2_O_3_-Au NP nano composites	Glucose solution	GOx	0.5	ABTS	7.0	-	188
Colorimetry	Au@BSA NPs-GO nano composites	Glucose solution	GOx	0.6	TMB	4.0	-	189
Colorimetry	HRP·H_2_O_2_·TMB	Urine	GOx	0.03	TMB		-	190
Colorimetry	AuNPs 2,2′-azino-bis(3-ethylbenzothiazoline-6-sulfonic acid) radical (ABTS+•)	Glucose solution	GOx	80	TMB	4.0	-	14
Colorimetry	Carboxyl-NS@GOx	Urine	GOx	125	-	4.3	2	90
Colorimetry	C/H-Aerogel	Blood, sweat	GOx	11.4			10	191
Colorimetry	MGCN-chitin-AcOH	Blood, urine		0.055			3	192
Colorimetry	MnO_2_ nano-oxidizers	Human blood		10			50	191
Colorimetry	Pt NPs	Glucose Uric acid		4	TMB + 4AD	7.0	20	182
Colorimetry	NL-MnCaO_2_	Human blood		23.86			-	193
Colorimetry	Fe-N-C/MgO	Glucose solution	GOx	2.1	TMB	4.0	-	194
Colorimetry	Pt^2+^_2.30_@g-C_3_N_4_	Human blood		0.01			-	195
Colorimetry	g-C_3_N_4_	Human serum	GOx	0.71	TMB	5.0	-	196
Colorimetry	Pd91-GBLP NPs	Human blood		1			-	197
Colorimetry	Fe_3_O_4_@MnO2	Uric acid Human plasma	GOx	0.27	TMB	-	1	66
Colorimetry	AuNCs	Human serum	GOx	74.7	TMB	3.0	-	198
Colorimetry	P-Co_3_O_4_	Human blood		0.69			30	199
Colorimetry	R-Co_3_O_4_	Human blood		0.32			30	199
Colorimetry	Fe_3_O_4_ magnetic nanoparticles (MNPs)	Glucose solution	GOx	30	ABTS	4.0	10	200
Colorimetry	Positively charged AuNPs	Glucose solution		4	TMB	4.0	15	201
Colorimetry	AuNPs	Urine	GOx	0.043		7.0	-	61
Colorimetry	ceria nanoparticles (CeO_2_ NPs)	Human blood	GOx	3	TMB	4.0	30	202
Colorimetry	Carbon nanodots (C-dots)	Human blood		0.4	TMB	3.5	15	203
Colorimetry	Ag nanoplates	Human blood	GOx	0.2		7.38	15	204
Colorimetry	Chitosan stabilized silver nanoparticles (Ch-Ag NPs)	Human blood	GOx	0.1		3.0	10	16
Colorimetry	DNA-embedded core-shell Au@Ag nanoparticles	Fetal bovine serum	GOx	0.01		4.5	30	205
Colorimetry	Nitrogen-doped graphene quantum dots	Serum	GOx	16	TMB	3.0	90	206
Colorimetry	V_2_O_3_-OMC	Serum	GOx	3.3	ABTS	4.0	10	207

**Table 7:** The summary of recent machine learning based colorimetric analysis.

Learning type	Purpose	Image format	Machine learning model	Software	Training data	Color space	Sample	Ref
Supervised learning	Classification	RAW JPEG	Least-Squares Support-Vector Machine (LS-SVM)	MATLAB	385 images	RGB, HSV, LAB	Hydrogen peroxide	208
Supervised learning	Classification	JPEG	Linear Discriminant analysis (LDA), Gradient Boosting Classifier (GBC), Random forest RF)	Python, MATLAB	224 images	RGB, HSV, LAB	Artificial Saliva	94
Supervised learning	Classification	JPEG	LDA, SVM, ANN	MATLAB, Python, Android studio	-	RGB, HSV, YUV, Lab	Alcohol solution	209
Supervised learning	Classification	JPEG	LDA Ensemble bagging classifier (EBC)	Matlab, Android studio	616 images	RGB, HSV, YUV, LAB	Artificial saliva	210
Supervised learning	Classification	JPEG	Convolutional neural network (CNN)	MATLAB	1600 images	RGB	Glucose solution	211
Supervised learning	Classification	Spectrum	Support vector machine-radial basis function (SVM-RBF)	-	-	-	Glucose solution	212
Supervised learning	Classification	JPEG	Multi-Layer Perceptron (MLP), Residual Network (ResNet), CNN	-	490 images	RGB	C-reactive protein (CRP)	212
Supervised learning	Classification	JPEG, RAW	LS-SVM	MATLAB	450 images	RGB	prepared PH solution	213
Supervised learning	Classification	JPEG, Spectrum	Faster region-based (CNN)	-	1500 images	RGB	Urine	102
Supervised learning	Classification	RAW	Artificial neural networks (ANNs)	MATLAB	160 and 54 data points	CMYK	Artificial urine	62
Supervised learning	Classification	NIR Spectrum	Deep neuronal network (DNN)	-	1024 dataset	-	Serum glucose	39
Supervised learning	Classification	Spectrum	Multi-Channel -CNN	-	-	-	Glucose solution	214

## Abbreviations

ISF: interstitial fluid
LOD: limit of detection
NIRS: near-infrared reflectance spectroscopy
OCT: optical coherence tomography
NIR: near-infrared
MVC: multivariate calibration
GBPs: glucose biding proteins
QDs: quantum dots
FRET: fluorescence resonant energy transfer
OP: optical polarimetry
PAS: photoacoustic spectroscopy
SNR: signal-to-noise ratio
RI: reverse iontophoresis
GOx: glucose oxidase
EIS: electrical impedance spectroscopy
CMOS: complementary metal-oxide-semiconductor
CCD: charge couple device
NPs: nanoparticles
AuNPs: gold nanoparticles
RGB: red, green, and blue
CMYK: cyan, magenta, yellow, and black
HSB: hue, saturation, and brightness
CeO_2_: cerium oxide
μPADs: microfluidic paper-based devices
cTnl: cardiac troponin I
MOF: metal-organic framework
HRP: horseradish peroxidase
TMB: tetramethylbenzidine
g-C3N4: graphitic carbon nitride
rGO: graphene oxide
HA-DA: dopamine-functionalized hyaluronic acid
g-C3N4:GCN: graphitic carbon nitride
GBMP: glucose-biosensing microneedle patch
GOx-MnO_2_@GO: GOx-conjugated MnO_2_/graphene oxide nanozymes
MeGel: methacrylate gelatin
CNPs: cerium oxide nanoparticles
NECL: nanoparticle-embedded contact lens
PEG: polyethylene glycol
HUVECs: human umbilical vein endothelial cells
HCECs: human corneal epithelial cells
LS-SVM: Least-Squares Support-Vector Machine
LDA: Linear Discriminant analysis
GBC: Gradient Boosting Classifier
RF: Random forest
EBC: Ensemble bagging classifier
SVM-RBF: Support vector machine-radial basis function
CNN: Convolutional neural network
MLP: Multi-Layer Perceptron
ResNet: Residual Network
CuBDC: copper 1,4-benzenedicarboxylate, copper-doped carbon-based nanozyme
PBNS: Prussian blue nanoparticles
WSNF: nanohybrid fiber
